# Circ-RAPGEF5 promotes intrahepatic cholangiocarcinoma progression by stabilizing SAE1 to facilitate SUMOylation

**DOI:** 10.1186/s13046-023-02813-y

**Published:** 2023-09-13

**Authors:** Junhao Zheng, Yali Wang, Liye Tao, Jingwei Cai, Zefeng Shen, Yang Liu, Haoyu Pan, Shihao Li, Yeling Ruan, Tianyi Chen, Zhengtao Ye, Kainan Lin, Yin Sun, Junjie Xu, Xiao Liang

**Affiliations:** 1 Key Laboratory of Laparoscopic Technology of Zhejiang Province, Department of General Surgery, Sir Run-Run Shaw Hospital, Zhejiang University School of Medicine, Hangzhou, 310016 China; 2 Zhejiang Minimal Invasive Diagnosis and Treatment Technology Research Center of Severe Hepatobiliary Disease, Zhejiang Research and Development Engineering Laboratory of Minimally Invasive Technology and Equipment 310016, Hangzhou, China; 3 Zhejiang University Cancer Center, Hangzhou, 310058 China; 4 Liangzhu Laboratory, Zhejiang University Medical Center, Hangzhou, 311121 China; 5 Department of Radiation Oncology, University of Rochester Medical Center, Rochester, NY 14642 USA

**Keywords:** Intrahepatic cholangiocarcinoma, Circ-RAPGEF5, SAE1, SUMOylation, AKT

## Abstract

**Background:**

Intrahepatic cholangiocarcinoma (ICC) is an aggressive malignancy with a poor prognosis. The underlying functions and mechanisms of circular RNA and SUMOylation in the development of ICC remain poorly understood.

**Methods:**

Circular RNA hsa_circ_0001681 (termed Circ-RAPGEF5 hereafter) was identified by circular RNA sequencing from 19 pairs of ICC and adjacent tissue samples. The biological function of Circ-RAPGEF5 in tumor proliferation and metastasis was examined by a series of in vitro assays. A preclinical model was used to validate the therapeutic effect of targeting Circ-RAPGEF5. RNA pull-down and dual-luciferase reporter assays were used to access the RNA interactions. Western blot and Co-IP assays were used to detect SUMOylation levels.

**Results:**

Circ-RAPGEF5, which is generated from exons 2 to 6 of the host gene RAPGEF5, was upregulated in ICC. In vitro and in vivo assays showed that Circ-RAPGEF5 promoted ICC tumor proliferation and metastasis, and inhibited apoptosis. Additionally, high Circ-RAPGEF5 expression was significantly correlated with a poor prognosis. Further investigation showed that SAE1, a potential target of Circ-RAPGEF5, was also associated with poor oncological outcomes. RNA pull-down and dual-luciferase reporter assays showed an interaction of miR-3185 with Circ-RAPGEF5 and SAE1. Co-IP and western blot assays showed that Circ-RAPGEF5 is capable of regulating SUMOylation.

**Conclusion:**

Circ-RAPGEF5 promotes ICC tumor progression and SUMOylation by acting as a sponge for miR-3185 to stabilize SAE1. Targeting Circ-RAPGEF5 or SAE1 might be a novel diagnostic and therapeutic strategy in ICC.

**Supplementary Information:**

The online version contains supplementary material available at 10.1186/s13046-023-02813-y.

## Background

Intrahepatic cholangiocarcinoma (ICC) is a malignancy that originates from the epithelial cells of the hepatic bile ducts. Hepatocellular carcinoma (HCC) is the most common type of primary liver cancer followed by ICC, which accounts for 10% to 15% of all primary liver cancers [1, 2]. The incidence of ICC varies among different regions, races, and sexes. The Asian population has a higher incidence of ICC than that in the European and American populations [3]. In recent years, there has been a considerable increase in the incidence of ICC [4]. Despite advances in diagnosis and treatment of ICC, it remains a challenging disease with a poor prognosis because of its early onset of tumor invasiveness, high recurrence rates, and propensity for distant metastasis [5]. Therefore, identifying new molecular targets is crucial to a better understanding of the pathogenesis and tumor progression of ICC.

Circular RNAs are a class of non-coding RNAs characterized by their unique loop-like structures resulting from the back-splicing of exons or introns from pre-mRNAs [6, 7]. These peculiar structures render them resistant to nucleic acid exonucleases and confer enhanced stability compared with linear RNAs [8]. Circular RNAs play a crucial role in tumorigenesis [6]. Circular RNAs possess diverse and critical regulatory functions, including acting as molecular sponges for microRNA (miRNA) and protein to modulate transcription [9–11], interfering with pre-mRNA normal splicing to regulate host gene mRNA translation [12–14], and forming circular RNA–protein complexes to compete with mRNAs for protein binding [15–17]. Recent studies have also shown that circular RNAs can serve as templates for encoding functional small peptides, thereby regulating essential biological processes, such as metabolism reprogramming [18], tumor cell proliferation, and aggressiveness [19, 20]. Circular RNA has a stable and conserved nature. Therefore, circular RNA has great potential as a reliable biomarker for the diagnosis and prognosis of tumors, and may be a promising therapeutic target in oncology. Numerous recent studies have further highlighted the crucial role of circular RNAs as novel regulatory RNA molecules in the development of ICC [21–26].

SUMOylation is a crucial cellular process in which a small ubiquitin-like modifier (SUMO) protein covalently modifies some target proteins by forming an ester bond [27]. This protein modification regulates many cellular processes, such as gene expression, DNA damage repair, protein localization, the cell cycle, regulation of transcription factor activity, and apoptosis [28]. SUMO is an approximately 12-kDa protein that is widely found in eukaryotes. The target protein of SUMOylation comprises transcription factors, nuclear pore complexes, and cytoplasmic proteins. SUMOylation can modulate the activity, stability, subcellular localization, and interactions of target proteins [27]. SUMOylation plays an essential role in tumorigenesis and development by regulating the function and expression of various tumor-related proteins. The expression of SUMOylation cascade proteins, such as SENP, SUMO-activating enzyme subunit 1 (SAE1)/2, UBC9, and E3 ligases, is upregulated in different types of cancer [28]. Among them, SAE1 is an E1-like protein and is involved in the development and progression of several cancers, such as breast, liver, and pancreatic cancer [29–31]. SAE1 inhibitors have been suggested as a potential therapeutic target because they can interfere with the growth and spread of tumor cells [31, 32]. However, the precise role and relevance of SUMOylation and SAE1 regulation mechanisms in ICC have not been fully determined.

In this study, we identified a novel circular RNA, Circ-RAPGEF5 (hsa_circ_0001681), which is significantly upregulated in ICC and is correlated with poor survival of patients. Our findings shed light on a previously unknown regulatory mechanism involving Circ-RAPGEF5 and suggest its potential as a therapeutic target for ICC. Our study shows that Circ-RAPGEF5 promotes ICC cell proliferation, migration, and invasion by enhancing SUMOylation within ICC cells. Mechanistically, Circ-RAPGEF5 acts as a sponge for miR-3185 to increase SAE1, which is a major protein involved in SUMOylation. Additionally, Circ-RAPGEF5 can enhance AKT SUMOylation, leading to tumorigenesis and progression of ICC.

## Materials and methods

### Patients and tissue samples

Paired ICC tumor tissues and adjacent normal tissues were collected from 110 ICC patients who underwent surgical therapy at Zhejiang University, School of Medicine, Sir Run Run Shaw Hospital (Hangzhou, China) between January 2014 and December 2019. Among them, 19 pairs were performed circular RNAs sequencing. For circular RNAs sequencing, the total RNAs were first treated with ribo-zero-magnetic-kit (Epicentre) and RNase R (Epicentre) to remove the liner RNAs and Ribosomal RNAs. The sequencing libraries were generating using NEBNext® UltraTM RNA Library Prep Kit for Illumina® (NEB, USA). Then the treated sample were sequenced on an Illumina Hiseq platform generating paired-end reads of 125 bp/150 bp length. This sequencing initiative was conducted by Novogene (Beijing, China). The sequencing data have been uploaded to the NGDC republic database (https://ngdc.cncb.ac.cn/) under accession number PRJCA018197.

The remaining 91 pairs were manufactured to tissue microarrays to validate clinical outcomes. The expression levels were assessed by percentage of stained cells (0–100%) and the staining intensity (0 = negative, 1 = weak, 2 = moderate, 3 = strong). Semi-quantitative assessments were conducted using histochemistry score (H-SCORE), which were computed as the multiplication of the percentage of stained cells (ranging from 0 to 100%) and the staining intensity (ranging from 0 to 3). All H-scores were standardized to a range of 0 to 100. Scores equal to or less than 50 were considered as low expression, while scores above 50 were regarded as high expression. The study was approved by the Ethics committee of Sir Run Run Shaw Hospital.

### Cell culture and transfections

The human ICC cell lines (RBE, CCLP1, 9810, HUCCT1) and human intrahepatic biliary epithelial cell (HIBEC) were obtained from the American Type Culture Collection (ATCC, Manassas, VA). The RBE, CCLP1, and HUCCT1 cell lines were maintained in high glucose Dulbecco’s Modified Eagle’s Medium (DMEM, Gibco, USA), containing with 10% fetal bovine serum. The 9810 and HIBEC cell lines were maintained in RPMI 1640 (Gibco, USA), supplemented with 10% fetal bovine serum. All the cell lines were cultured in a humidified incubator at 37 °C with 5% CO2. We constructed the Circ-RAPGEF5 overexpression plasmid through homologous recombination using the pLC5-ciR vector (Geneseed Biotech, Guangzhou, China). The siRNA and plasmid transfection were performed using Lipofectamine 3000 reagent (Thermo Fisher Scientific, Waltham, MA) according to the manufacturer's instructions. The transcriptome sequencing analysis were performed by RiboBio Biotechnology (Guangzhou, China). Briefly, RBE, CCLP1 and 9810 cells were transfected with Si-Circ-RAPGEF5 or the negative control. Subsequently, total RNAs were extracted from these cells using TRIzol reagent (Invitrogen, CA, USA). After constructing sequencing libraries, the samples underwent paired-end 150 bp sequencing on an Illumina Novaseq™ 6000 platform. The sequencing data have been uploaded to the NGDC republic database (https://ngdc.cncb.ac.cn/) under accession number PRJCA018197.

### Functional assays in vitro

Cell viability was examined by Cell Counting Kit (CCK-8) assay (YEASEN, Shanghai, China). In detail, 0.5 ~ 1.0×10^4^ cells suspended in 100 µl of complete culture media were seeded in 96-well plates with three replicates. The absorbance at the wavelength of 450 nm was detected after adding 10 µL of CCK-8 solution into each well and incubating at 37 °C for 90 min.

Cells were seeded in the 6-well plate for colony formation assays at a density of 0.5 ~ 1.5×10^3^ per well. After culturing for two weeks, cells were fixed with 4% paraformaldehyde and stained with 0.1% crystal violet.

The cell proliferation ability was measured by the EdU labeling assay using a Yefluor 594 Edu Imaging Kits (YEASEN, Shanghai, China) following the manufacturer's instructions.

The transwell assay was performed to examine the cell migration and invasion ability. The eight µm-pore size membranes transwell chamber (Transwell™ Permeable Polyester Membrane Inserts from Corning Inc.) coated with or without Matrigel was used for the invasion and migration ability test. The transfections treated cells (2–6×10^4^, according to the state of cell growth) were added into the upper chamber with 150ul FBS-free medium, and 600ul 10% FBS-supplemented medium was added into the underlying Chamber. After being maintained for 24 or 48 h, the cells migrated to the bottom surface of the chamber and were fixed with 4% paraformaldehyde, stained with 0.1% crystal violet, and pictured by microscope. All cells were inoculated with three duplicate chambers. The cell counting was conducted using ImageJ software (National Institutes of Health, USA).

### Flow cytometry assay of cell cycle and apoptosis

For cell cycle assay, transiently transfected cells were trypsinized with Trypsin/EDTA (0.25%) (Gibco by Life Technologies, Grand Island, NY, USA) and fixed in 75% alcohol overnight at -20 °C after PBS washed. We used a Cell Cycle Staining Kit (MUTLI SCIENCES, Hangzhou, China) for propidium iodide (PI) staining.

For the apoptosis test, treated cells after trypsinization were stained by Annexin V-APC/7-AAD apoptosis kit (MUTLI SCIENCES, Hangzhou, China).

The cell cycle distribution percentage and the apoptotic rate were analyzed by the BDLSRII flow cytometer (MUTLI SCIENCES, Hangzhou, China).

### In vivo studies

We use a patient-derived xenograft (PDX) to construct subcutaneous xenograft models. The tumor tissue was obtained from a 59-year-old male who underwent radical resection at Zhejiang University, School of Medicine, Sir Run Run Shaw Hospital. Informed consent was obtained from the patient, and the study protocol was approved by the Sir Run Run Shaw Hospital Institution Review Board. We implanted the fresh ICC tumor blocks removed from the patient into the subcutaneous space of 4-week-old nod/scid mice. Two months later, when the tumor had grown, we released the first-generation PDX and disaggregated it into 1mm^3^ tissue blocks. The small tissue blocks were subcutaneously implanted into 4- to 6-week-old male BALB/c nude mice to establish the second-generation PDX model. When the tumors grow to 3 mm in diameter, we peritumorally inject with sh-Circ-RAPGEF5 lentiviruses and control lentiviruses every two days. Mice were euthanized, and the tumor was isolated for analysis after 16 days’ treatment.

For tumor metastasis models, we constructed lentivirus containing short hairpin RNAs (shRNAs) cell. 5 × 10^6^ stably transfected RBE-sh-NC-luc and RBE-sh-RAPGEF5-luc cells suspended in 100 µL PBS were injected into the tail vein of 4- to 6-week-old male NOD/SCID mice. Three months later, tumor metastasis was scanned by the IVIS system following intraperitoneal injection of 150 mg/Kg D-Luciferin.

The animal studies were subjected to the Association for the Assessment and Accreditation of Laboratory Animal Care and the Institutional Animal Care and Use Committee guidelines.

### Quantitative real-time PCR (qRT-PCR), western blot, and immunohistochemical (IHC)

The total RNA of tissues and cells was extracted using TRIzol Reagent (Invitrogen, Carlsbad, CA). 1 ug of total RNA was transcribed into complementary DNA using Hifair II 1st Strand cDNA Synthesis Kit (YEASEN, Shanghai, China). Quantitative real-time PCR (qRT-PCR) was performed using SYBR Green Master Mix (YEASEN, Shanghai, China) and detected by LightCycler 480 (Roche, Basel, Switzerland). The relative expression of RNA was normalized by glyceraldehyde-3-phosphate dehydrogenase (GAPDH). Primers used in this study are displayed in Table S2.

For western blot analysis, protein of tissue and cells were extracted using RIPA Lysis Buffer (Beyotime, Shanghai, China) containing protease inhibitor cocktail (Thermo Fisher Scientific, USA). Proteins were separated on 10–12% SDS/PAGE gel and then transferred onto PVDF membranes (Millipore, Billerica, MA). After blocking and incubating by corresponding antibody and horseradish peroxidase (HRP)-conjugated secondary, the blots were visualized by the ECL system (Thermo Fisher Scientific, Rochester, NY). Antibodies and reagents used in this study are shown in Table S3. We conducted three independent replicate experiments for all western blot analysis (Figure S4-8).

For H&E and immunohistochemical (IHC) staining, tumor tissues were fixed in 4% paraformaldehyde and cut into 3 µm sections after paraffin-embedded. The slides were blocked with 5% goat serum and incubated with SAE1 antibodies overnight. On the second day, we used the GTvision immunohistochemistry kit to visualize the staining following the manufacturer’s protocol.

### Fluorescence in situ hybridization (FISH) and immunofluorescence

We used an RNA FISH kit (RiboBio Biotechnology, Guangzhou, China) to explore the subcellular location of Circ-RAPGEF5 and the colocalization of Circ-RAPGEF5 and miR-3185. The CY3-conjugated probe against 18S and Circ-RAPGEF5 and FAM-conjugated probes against miR-3185 were synthesized by RiboBio. The specific operation of the FISH assay could refer to the manufacturer’s instructions for the RNA FISH kit. The FISH probe sequence in this study can be found in Table S4.

An immunofluorescence assay was performed to detect the expression level of SAE1 in patients’ specimens and tissue microarrays. The tissue slide fixed with 4% formaldehyde was blocked with 5% goat serum and incubated with SAE1 antibody. The Nuclei were counterstained with DAPI. The fluorescence pictures were visualized by EVOS (Thermo Fisher Scientific, Waltham, MA).

### RNA pull-down and coimmunoprecipitation assay (Co-IP)

We used streptavidin magnetic beads (Thermo Fisher Scientific, Waltham, MA) for RNA pull-down assay. The biotin-labeled Circ-RAPGEF5 probe, miR-3185 probe, and corresponding negative control probe were synthesized by RiboBio (RiboBio Biotechnology, Guangzhou, China). The beads were washed with RNase-free lysis buffer and blocked with BSA for preparation. The blocked beads were incubated with a biotinylated target, and the negative control probe and cell lysis contained at least 1×10^7^ cells at 4 °C for one h. The RNA pull-down complexes were used to purify RNA by the TRIzol reagent. The enrichment of Circ-RAPGEF5 was detected by qRT-PCR. The biotin-probe sequence in this study can be found in Table S5.

Due to the large number of cells required for Co-IP, we used stable lentivirus-transfected RBE cells for IP. Co-IP was performed with the Protein A/G Agarose Beads (Santa Cruz) according to the protocol. Lysis buffer rewashed A/G agarose beads were incubated were cell lysates and 2ug of antibodies at 4°C overnight. The antibody complex was centrifugally washed three times, and western blot analysis was used to detect the binding effect of the corresponding proteins.

### Dual-luciferase reporter assays

The 3’UTR (untranslated region) of SAE1 containing the potential binding site and mutational binding site of miR-3185 was synthesized by TSINGKE Biotechnology (Beijing, China). The synthesized 3’UTR was inserted into a pmirGLO plasmid containing the firefly luciferase gene and the Renilla luciferase gene. The 3’UTR was placed at the tail of the firefly luciferase gene. ICC cells transfected with SAE1 3’UTR plasmid were then transfected with Mimic-miR3185 and Mimic-NC. Five days later, we used the Promega Dual-Luciferase Reporter assay system (Promega, USA) to detect the fluorescence intensity of the firefly luciferase and Renilla luciferase.

### Statistical analysis

The data were displayed mean ± standard error in at least three duplicated independent experiments. T-test was used for comparison between the two groups. One-way analysis of variance was used for comparisons between the means of multiple groups. The count data were described as absolute logarithms, and the chi-square test or Fisher exact probability method was used for comparison between groups. We used the Kaplan–Meier method to evaluate cumulative survival time and analyzed the comparisons with a log-rank test. A multivariate Cox regression model was performed to identify independent prognostic factors. The correlation between Circ-RAPGEF5 and SAE1 expression was analyzed using Pearson’s correlation test. A hypothetical model diagram was drawn by Figdraw (http://figdraw.com). *P* < 0.05 was considered statistically significant. (*P < 0.05, **P < 0.01, and ***P < 0.001). The statistic of the comparison was calculated using GraphPad Prism 9 Software (San Diego, CA).

## Results

### Identification and characteristics of Circ-RAPGEF5 in ICC

**Fig. 1:**
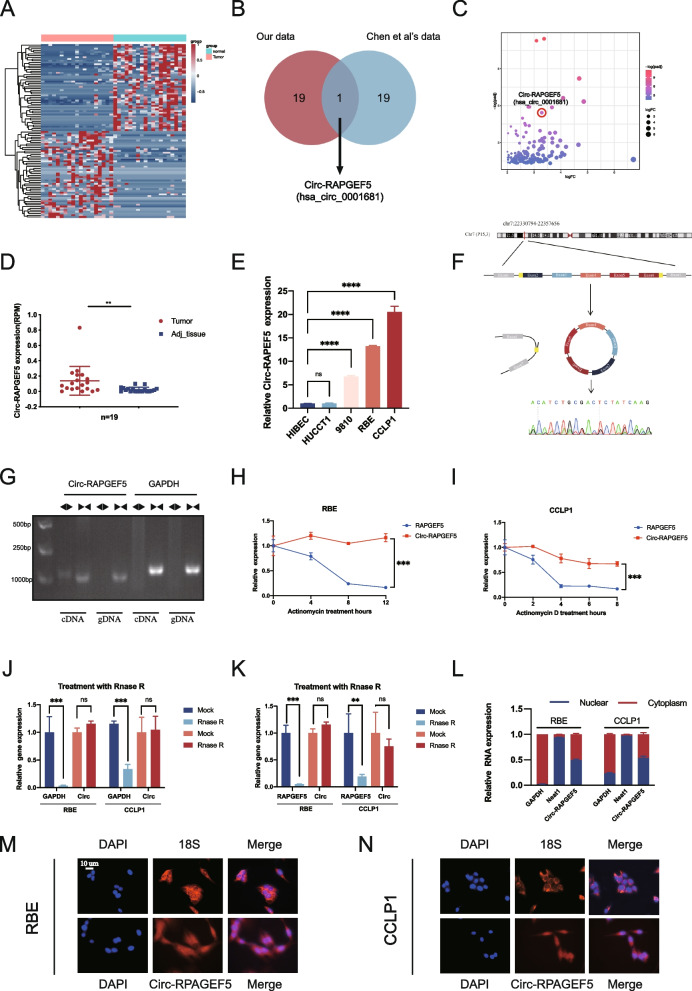
Circ-RAPGEF5 identification and validation in ICC cells and tissues. **A** Heatmap of the top 50 upregulated and downregulated expression circular RNAs between ICC tissues and adjacent normal tissues according to the bulk RNA sequencing of 19 patients. **B** Venn diagram illustrating the overlap of top 20 upregulated expression circular RNAs in our data and Chen et al.’ s data, only Circ-RAPGEF5 was identified. **C** Volcano plot of upregulated expressed circular RNAs from 19 paired samples. **D** Relative Circ-RPAGEF5 expression in 19 ICC tumor tissues and matched adjacent normal tissues. **E** qRT-PCR analysis of the relative expression levels of Circ-RAPGEF5 in a panel of four human ICC cell lines (HUCCT1, 9810, RBE, and CCLP1) and a normal biliary epithelial cell line, HIBEC. **F** The schematic illustration showed the back-splicing of Circ-RAPGEF5, and Sanger sequencing validated the splicing site. **G** Circular formation of Circ-RAPGEF5 was confirmed through PCR and agarose gel electrophoresis using divergent and convergent primers in both gDNA and cDNA samples of RBE. GAPDH was used as a negative control. **H-I** qRT-PCR analysis of Circ-RAPGEF5 and linear RAPGEF5 expression levels after Actinomycin D treatment in RBE and CCLP1. **J-K** qRT-PCR analysis of Circ-RAPGEF5 compared to linear GAPDH and RAPGEF5 expression levels after RNase R treatment in RBE and CCLP1. **L-N** Subcellular fractionation and FISH assays indicated that Circ-RAPGEF5 was localized in both the cytoplasm and nucleus of RBE and CCLP1. All data are presented as the means ± SD of three independent experiments. *p < 0.05, **p < 0.01, ***p < 0.001, ****p < 0.0001

To identify the major circular RNAs involved in the progression of ICC, we performed bulk RNA sequencing of 19 pairs of tumor and adjacent tissue samples from patients with ICC in our center. A total of 262 differentially expressed circ RNAs were found. A heat map showed the top 50 upregulated and downregulated circular RNAs (Fig. 1A). We used external data to validate our sequencing results and found that Circ-RAPGEF5 (CircBase ID: hsa_circ_0001681) was significantly upregulated as shown by our results and sequencing data from Chen et al. [21] (Fig. 1B), which suggested that Circ-RAPGEF5 was associated with the development of ICC. A volcano plot showed the rank of Circ-RAPGEF5 in the differentially expressed circular RNAs from our 19 paired samples (Fig. 1C). Circ-RAPGEF5 was significantly upregulated in 19 ICC tumor tissues compared with adjacent paired normal tissues (Fig. 1D). Similarly, Circ-RAPGEF5 was significantly upregulated in ICC cell lines (9810, RBE, and CCLP1) compared with the normal biliary epithelial cell line (HIBECs) (Fig. 1E). Therefore, we selected Circ-RAPGEF5 for further in-depth study. Circ-RAPGEF5 is generated from exons 2 to 6 of the host gene RAPGEF5 located on chromosome 7 with a length of 516 nucleotides. We performed Sanger sequencing to determine the back-splicing site of Circ-RAPGEF5 (Fig. 1F). To confirm the circular structure of Circ-RAPGEF5, divergent primers and convergent primers were designed to amplify linear and back-splicing PCR products. We found that the convergent primers of Circ-RAPGEF5 and GAPDH amplified products of the expected size from cDNA and genomic DNA in ICC cells. Only the divergent primers of Circ-RAPGEF5, but not that of GAPDH, amplified PCR products from cDNA, but not from genomic DNA (Fig. 1G). In addition, qRT-PCR showed enhanced resistance of Circ-RAPGEF5 to RNase R and actinomycin D in RBE and CCLP1 cells compared with host gene RAPGEF5 mRNA (Fig. 1H–K). These results suggest that Circ-RAPGEF5 is a loop structure rather than a linear structure. We performed qRT-PCR to examine the abundance of Circ-RAPGEF in the nucleus and cytoplasm of ICC cells. Interestingly, Circ-RAPGEF5 was detected in the cytoplasm and nucleus of ICC cells (Fig. 1L), consistent with the FISH assay results (Fig. 1M, N).

Overall, we identified the circular RNA hsa_circ_0001681, termed Circ-RAPGEF5, which was highly expressed in ICC cell lines and tumor tissues. Circ-RAPGEF5 was widely distributed in the cytoplasm and nucleus of ICC cells.

### Circ-RAPGEF5 promotes the proliferation and metastasis of ICC in vitro

We designed and synthesized two siRNAs (Si-Circ-RAPGEF5#1 and Si-Circ-RAPGEF5#2) specifically for the Circ-RAPGEF5 back-splicing site and successfully constructed Circ-RAPGEF5 overexpression plasmids.

**Fig. 2:**
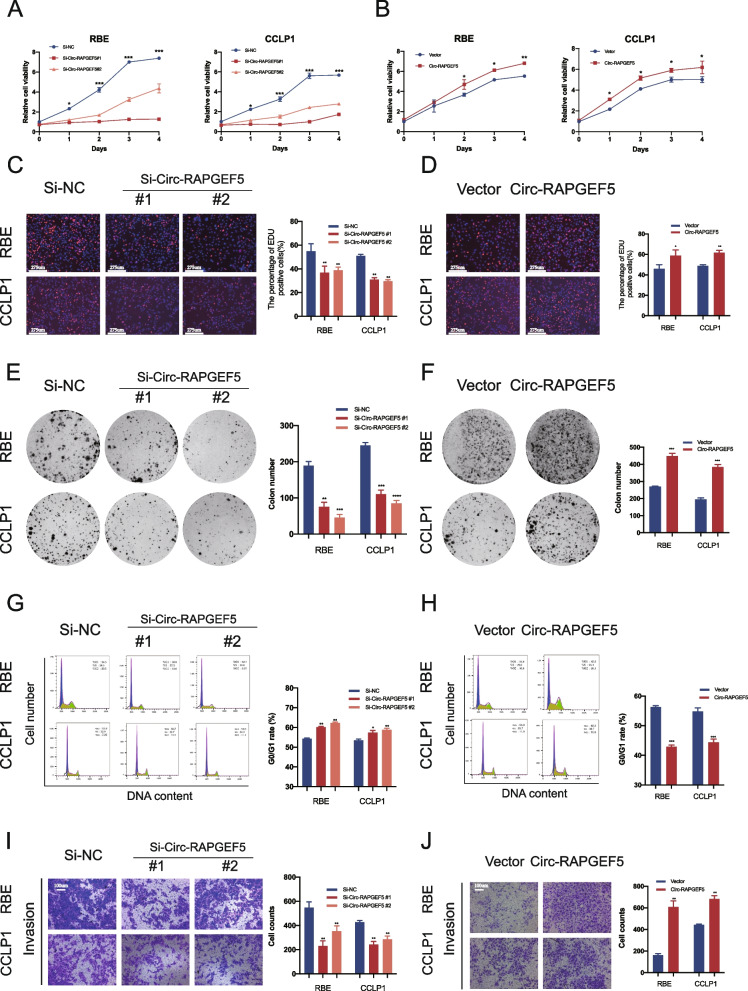
Effects of Circ-RAPGEF5 on the proliferation and invasion in vitro. **A-B** CCK8 assays to test cell growth viability of Circ-RAPGEF5 knockdown or overexpression in RBE and CCLP1 cells. **C-D** EdU labelling assays to test the cell proliferation ability. **E–F** Colony formation assays to evaluate cell long-term proliferation ability. **G-H** Cell cycle analysis detected by flow cytometry in Circ-RAPGEF5 knockdown or overexpression cells. **I-J** The invasion ability was assessed by Matrigel substrated transwell assay in Circ-RAPGEF5 knockdown or overexpression cells. All data are presented as the means ± SD of three independent experiments. *p < 0.05, **p < 0.01, ***p < 0.001, ****p < 0.0001

The two siRNAs against Circ-RAPGEF5 and Circ-RAPGEF5-overexpressed plasmids were transfected into RBE and CCLP1 cells. RAPGEF5 mRNA and protein expression of the host gene remained unchanged when Circ-RAPGEF5 was knocked down (Figure S1A, B). The efficacy of Circ-RAPGEF5 knockdown and overexpression was verified by qRT-PCR (Figure S1C–F). CCK-8, EdU labeling, and colony formation assays showed that Circ-RAPGEF5 silencing significantly inhibited cell proliferation in RBE and CCLP1 cells, while Circ-RAPGEF5 overexpression caused the opposite effect (Fig. 2A–F). Flow cytometry of the cell cycle showed that downregulation of Circ-RAPGEF5 inhibited the G1 to S transition of the cell cycle. However, Circ-RAPGEF5 overexpression significantly reduced the number of G0/G1 phase cells and significantly increased the number of S phase cells (Fig. 2G, H). The detection of apoptosis indicated that the apoptosis rate of siRNAs transfected RBE and CCLP1 cells was significantly increased. In contrast, the apoptosis rate of ICC cells was significantly reduced after Circ-RAPGEF5 overexpression (Figure S2A, B). In addition, the invasion (Fig. 2I, J) and migration (Figure S2C, D) capability test using the transwell assay showed that the loss of Circ-RAPGEF5 significantly impaired the migration and invasion of ICC cells, while overexpression of Circ-RAPGEF5 enhanced this ability.

### Circ-RAPGEF5 promotes the proliferation and experimental metastasis of ICC in vivo

**Fig. 3:**
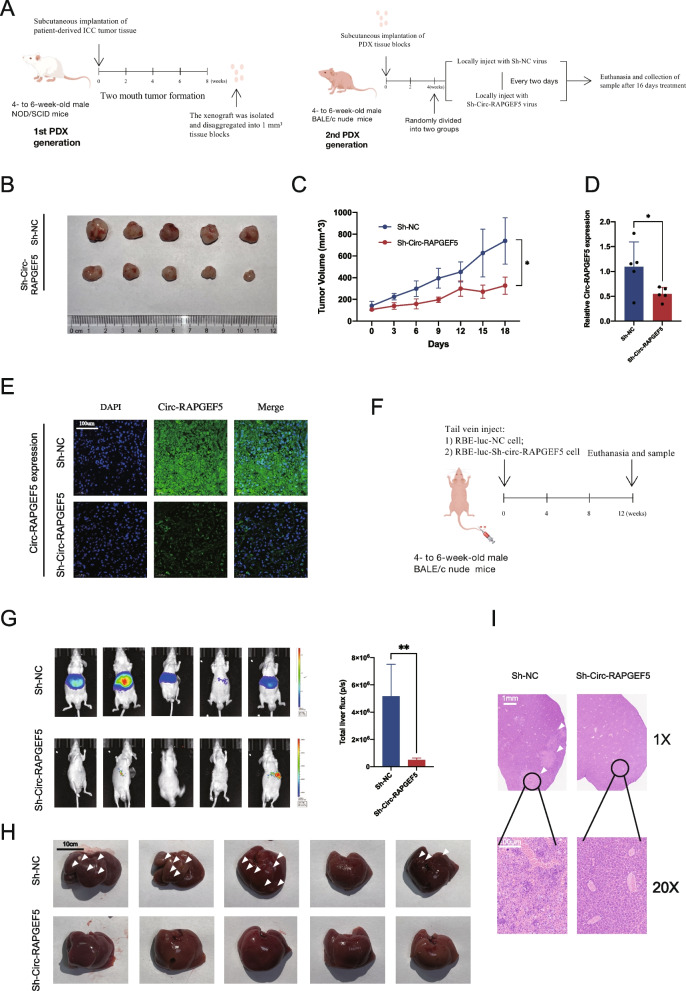
Effects of Circ-RAPGEF5 on the proliferation and metastasis in vivo. **A** NOD/SCID mice (4- to 6-week-old males) were subcutaneously implanted with ICC patient-derived xenograft (PDX). 2 months later, the xenograft was removed for a second round of axillae implantation. On week 4, the second-generation PDX mice were injected at the implantation site within Sh-Circ-RAPGEF5 or Sh-NC lentivirus every two days for 16 days. Animals were sacrificed, and the second-generation PDX was isolated on day 16. **B** Collection of subcutaneous xenografts of two groups. **C** Growth curve of xenografts in vivo measured every two days. **D** qRT-PCR analysis of the relative expression levels of Circ-RAPGEF5 in xenografts tissue of two groups. **E** The expression of Circ-RAPGEF5 in two groups detected by FISH (20x). **F** Liver metastasis models were established by injecting Sh-NC and Sh-Circ-RAPGEF5 RBE-luc cells via the tail vein. **G** Representative bioluminescent images and comparison of fluorescence intensity of liver metastasis. **H** Image of liver metastasis in two groups. The arrow indicates the metastatic lesions. **I** Representative H&E staining of liver metastatic lesions. All data are presented as the means ± SD. *p < 0.05, **p < 0.01, ***p < 0.001, ****p < 0.0001

To investigate the role of Circ-RAPGEF5 in the progression of ICC in a model that closely mimics the clinical situation, we selected a patient-derived tumor xenograft (PDX) model for the in vivo experiments. We first implanted fresh ICC tumor blocks excised from patients into the axillae of nod/scid mice on both sides. Approximately 2 months later, we removed and mashed the isolated tumors into 1 mm^3^ sections when the tumors grew large enough. The tumor tissues were reimplanted into the axillae of BALB/c nude mice. After 1 month of second-generation tumor growth, we started peri-tumor injections of lentivirus expressing sh-Circ-RAPGEF5 and negative control vector lentivirus every 2 days. After 16 days of treatment, we euthanized the mice (Fig. 3A). The tumor volume in the sh-Circ-RAPGEF5 treated group was significantly smaller than that in the control group (Fig. 3B, C). The qRT-PCR analysis and FISH assay of tumor tissues showed that sh-Circ-RAPGEF5 lentivirus treatment resulted in a significant reduction in Circ-RAPGEF5 expression (Fig. 3D, E). These results suggested that Circ-RAPGEF5 damage also inhibited tumor proliferation in vivo.

We also injected sh-Circ-RAPGEF5 stably transfected RBE-LUC cells and their control cells into the tail vein (Fig. 3F). After 3 months, obvious metastatic lesions in the liver were observed in the control group, and no obvious intrahepatic metastatic lesions were found in the sh-Circ-RAPGEF5 group (Fig. 3G, H, I). This result suggested that knocking down Circ-RAPGEF5 in ICC cells resulted in inhibition of their invasive and metastatic capabilities in vivo.

### Circ-RAPGEF5 promotes the progression of ICC by increasing the expression of SAE1

**Fig. 4:**
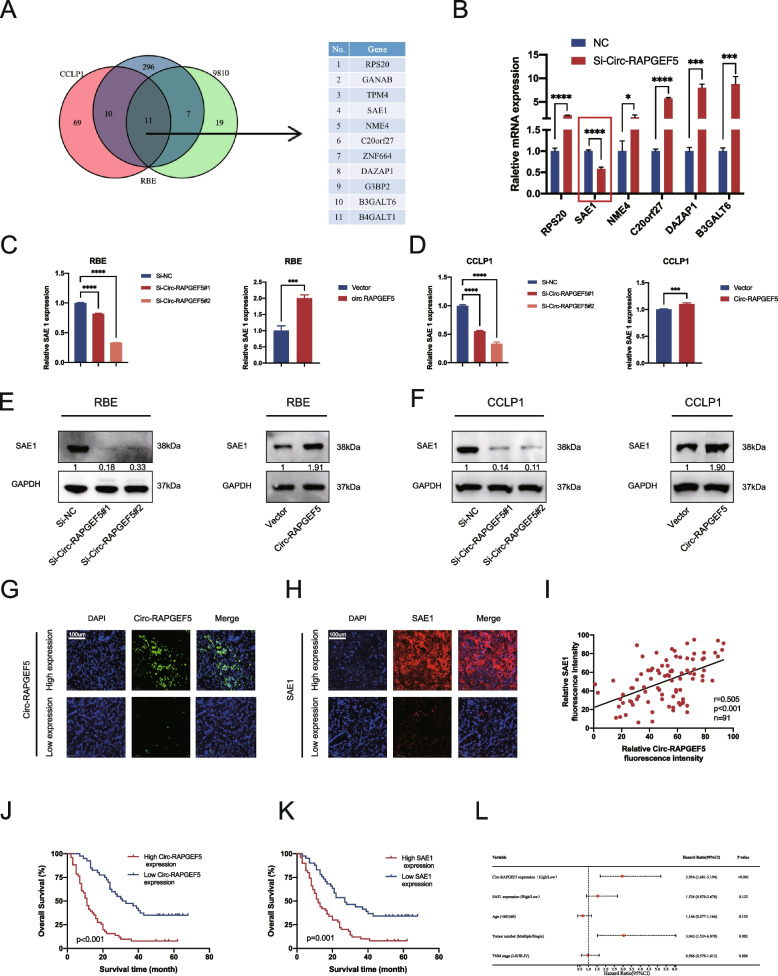
SAE1 might be a novel target of Circ-RAPGEF5. **A** Venn diagram of Circ-RAPGEF5 knockdown sequencing data in RBE, CCLP1, and 9810. **B** qRT-PCR analysis identified SAE1 was the only mRNA down-regulation after knockdown of Circ-RAPGEF5 **C-D** qRT-PCR analysis testing SAE1 mRNA expression after knockdown and overexpression of Circ-RAPGEF5 in RBE and CCLP1. **E–F** Western blot analysis accessed SAE1 protein level after changing the expression of Circ-RAPGEF5. **G-H** Representative FISH image for high and low expression of Circ-RAPGEF5 and SAE1 of the tissue microarray including 91 ICC patients' tumor tissues (20X). **I** Positive correlation between Circ-RAPGEF5 and SAE1 in 91 ICC patients' tumor tissues (*P* < 0.001). **J-K** Kaplan–Meier survival curves of patients with low or high Circ-RAPGEF5 and SAE1 expression based on tissue microarray. Survival comparison analysis was performed using the log-rank test. **L** Forest plot of multivariate COX analyses showing prognostic indicators for overall survival. *p < 0.05, **p < 0.01, ***p < 0.001, ****p < 0.0001

To investigate the specific mechanism by which Circ-RAPGEF5 promotes ICC cell invasion and migration, we performed transcriptome sequencing analysis of RBE, CCLP1, and 9810 cells transfected with Si-Circ-RAPGEF5 and negative controls. An analysis of the sequencing data showed that eleven mRNAs had significantly differential expression in the Si-Circ-RAPGEF5 group in all three cell lines compared with the negative control group (*P* < 0.05). Among them, six mRNAs were significantly downregulated, and five were significantly upregulated (Fig. 4A). We performed qRT-PCR to verify the six downregulated mRNAs and found that SAE1 was the most significantly downregulated mRNA after Circ-RAPGEF5 knockdown (Fig. 4B). This finding indicated that SAE1 expression may be closely related to Circ-RAPGEF5.

At the transcriptional and translation levels, we examined the consistent change characteristic of SAE1 in response to Circ-RAPGF5 (Fig. 4C–F). TCGA data also showed significantly higher expression of SAE1 in cholangiocarcinoma tissues compared to normal tissues. (Figure S3A) (http://gepia.cancer-pku.cn/). To further investigate the association between SAE1 and Circ-RAPGEF5, we performed the FISH assay in tissue microarrays containing 91 tumor tissues of patients with ICC. The patients were divided into high and low expression groups according to the expression levels of Circ-RAPGEF5 and SAE1 (Fig. 4G, H). We found that TNM staging was significantly worse in the Circ-RAPGEF5 high expression group than in the low expression group (*P* = 0.031). The TNM stage was also significantly advanced in the SAE1 high expression than in the low expression group (*P* = 0.018) (Tables 1 and 2). In addition, we found that SAE1 expression was significantly correlated with the expression of Circ-RAPGEF5 expression (Fig. 4I). A survival analysis showed that high expression of Circ-RAPGEF5 and SAE1 was significantly associated with a poor prognosis in patients with ICC (Fig. 4 J, K). Other public also confirmed a poor prognosis in patients with ICC in the group with high SAE1 expression (Figure S3B) [33]. qRT-PCR analysis and Immunohistochemistry showed low SAE1 expression in the Sh-Circ-RAPGEF5 virus-treated group of PDX (Figure S3C, D). A univariate Cox regression analysis showed that SAE1 and Circ-RAPGEF5 expression, the number of tumors, and the TMN stage were associated with the prognosis (Table S1). A forest plot of multivariate Cox regression showed that Circ-RAPGEF5 expression and the number of tumors were independent risk factors for the prognosis of patients with ICC in our cohort (Fig. 4L).

**Table 1:** Clinical characteristics of 91 ICC patients based on Circ-RAPGEF5 expression levels

Variables	All patients	Circ-RAPGEF5 expression		*p* value
		High expression (51)	Low expression (40)	
Age				0.632
< 60	25	13	12	
≥ 60	66	38	28	
Gender				0.501
Female	40	24	16	
Male	51	27	24	
Tumor number				0.915
Single	80	45	35	
Multiple	11	6	5	
Differentiation				0.722
Low	55	30	25	
Moderate/well	36	21	15	
Lymph node metastasis				0.180
Yes	39	25	14	
No	52	26	26	
Distant metastasis				0.364
Yes	15	10	5	
No	76	41	35	
TNM stage				0.031
I/II	43	19	24	
III/IV	48	32	16

**Table 2:** Clinical characteristics of 91 ICC patients based on SAE1 expression levels

Variables	All patients	SAE1		*p* value
		High expression (50)	Low expression (41)	
Age				0.412
< 60	25	12	13	
≥ 60	66	38	28	
Gender				0.993
Female	40	22	18	
Male	51	28	23	
Tumor number				0.537
Single	80	43	4	
Multiple	11	7	37	
Differentiation				0.443
Low	55	32	23	
Moderate/well	36	18	18	
Lymph node metastasis				0.503
Yes	39	23	16	
No	52	27	25	
Distant metastasis				0.001
Yes	15	14	1	
No	76	36	40	
TNM stage				0.018
I/II	43	18	25	
III/IV	48	32	16	

### SAE1 promotes the proliferation and metastasis of ICC

**Fig. 5:**
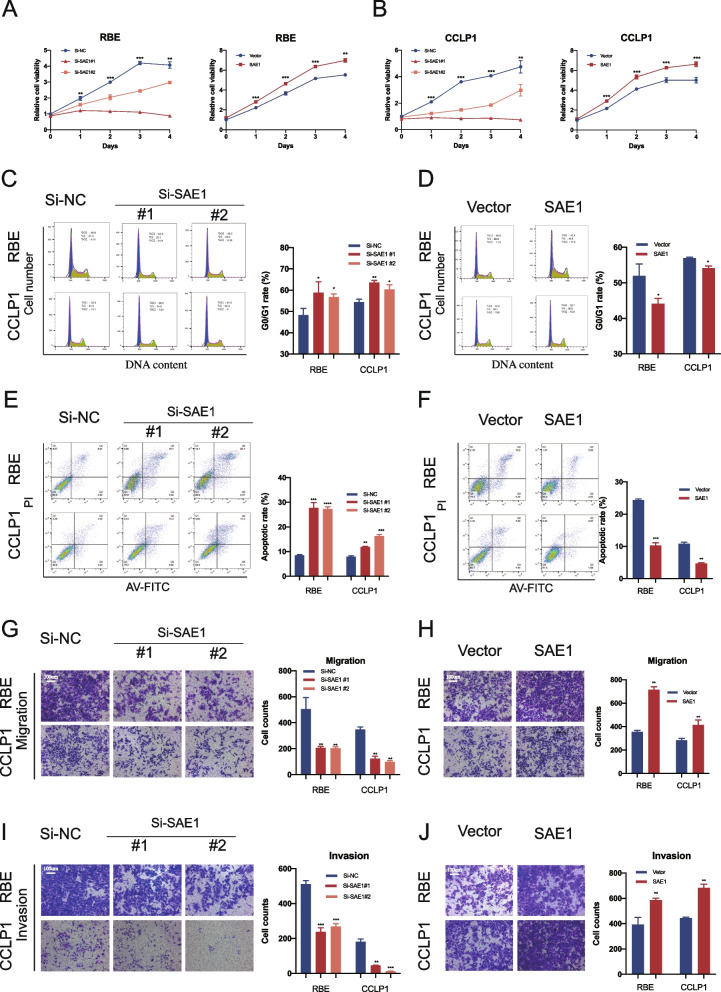
Effects of SAE1 on the proliferation and invasion in vitro. **A-B** CCK8 assays to test cell growth viability of SAE1 knockdown or overexpression RBE and CCLP1 cells. **C-D **Apoptosis rate detected by flow cytometry in SAE1 knockdown or overexpression cells. **E-F** Cell cycle detected by flow cytometry in Circ-RAPGEF5 knockdown or overexpression cells. **G-H** The migration ability of Circ-RAPGEF5 knockdown or overexpression RBE and CCLP1 were assessed by transwell assay. **I-J** The invasion ability was assessed by transwell assay. All data are presented as the means ± SD of three independent experiments. *p < 0.05, **p < 0.01, ***p < 0.001, ****p < 0.0001

The role of SAE1 in the progress of ICC has not been previously reported. Therefore, we investigated whether SAE1 expression affects the biological behavior of ICC cells. We designed two siRNAs (Si-SAE1#1 and Si-SAE1#2) and an SAE1 overexpression plasmid. The efficacy of SAE1 knockdown and overexpression was verified by qRT-PCR (Figure S1 E–J). We then examined the ICC cell proliferation and invasion ability on downregulation and overexpression of SAE1. The CCK-8 assay showed that knockdown of SAE1 significantly inhibited the proliferation of RBE and CCLP1 cells (Fig. 5A), while SAE1 overexpression significantly promoted the proliferation of RBE and CCLP1 cells (Fig. 5B). Flow cytometry of the cell cycle test showed that SAE1 knockdown significantly increased the phase of G0/G1 in RBE and CCLP1 cells, and inhibited the cell cycle transition to the S phase (Fig. 5C). In contrast, SAE1 overexpression showed the opposite effect of SAE1 knockdown (Fig. 5D). Flow cytometry of apoptosis showed that the downregulation of SAE1 significantly promoted cell apoptosis (Fig. 5E). In contrast, the proportion of apoptosis in ICC cells was significantly decreased when SAE1 was overexpressed (Fig. 5F). The migration and invasion ability tested by the transwell assay showed that SAE1 knockdown inhibited the migration and invasion of RBE and CCLP1 cells, while SAE1 overexpression significantly enhanced the migration and invasion of ICC cells (Fig. 5G–J). In conclusion, SAE1 significantly promoted proliferation, migration, and invasion and inhibited apoptosis in vitro.

### SAE1 overexpression alleviates the inhibitory effect of Circ-RAPGEF5 knockdown on tumor progression in vitro

**Fig. 6:**
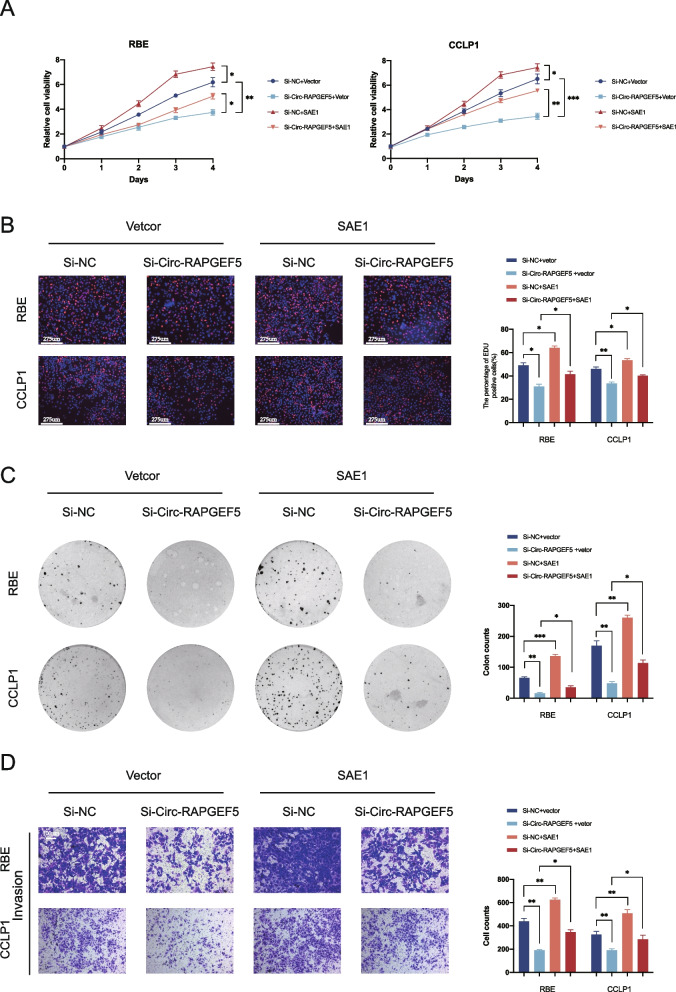
Circ-RAPGEF5 exerts its carcinogenic effects on ICC cells by promoting SAE1. **A** CCK-8 cell viability assay for RBE and CCLP1 cells transfected with Si-NC or Si-Circ-RAPGEF5 and vector or SAE1. **B** EdU labelling assays for RBE and CCLP1 cells transfected with Si-NC or Si-Circ-RAPGEF5 and vector or SAE1. **C** Colony formation assays for RBE and CCLP1 cells transfected with Si-NC or Si-Circ-RAPGEF5 and vector or SAE1.** D** Matrigel-coated transwell assays for RBE and CCLP1 cells transfected with Si-NC or Si-Circ-RAPGEF5 and vector or SAE1. All data are presented as the means ± SD of three independent experiments. *p < 0.05, **p < 0.01, ***p < 0.001, ****p < 0.0001

We used the SAE1 overexpression plasmid to investigate the role of SAE1 in the Circ-RAPGEF5-mediated occurrence and development of ICC. The CCK-8 cell viability and EDU labeling assays showed that Circ-RAPGEF5 knockdown significantly inhibited the proliferation of RBE and CCLP1 cells, while SAE1 overexpression significantly promoted the proliferation of ICC cells. Moreover, SAE1 overexpression alleviated the inhibitory effect of Circ-RAPGEF5 on ICC cell proliferation (Fig. 6A, B). Similar results were observed in the colony formation assay. SAE1 overexpression rescued the long-term inhibitory effect of Circ-RAPGEF5 knockdown on ICC cell proliferation (Fig. 6C). In addition, the Matrigel-coated transwell assay showed that SAE1 overexpression significantly reverse the inhibition by Circ-RAPGEF5 knockdown on ICC cell invasion in vitro (Fig. 6D). In summary, SAE1 rescued the inhibitory effect of Circ-RAPGEF5 on the proliferation and invasion of ICC cells in vitro. This finding suggests that SAE1 plays an important regulatory role in the Circ-RAPGEF5-mediated occurrence and development of ICC.

### Circ-RAPGEF5 upregulates SAE1 expression by sponging miR-3185

**Fig. 7:**
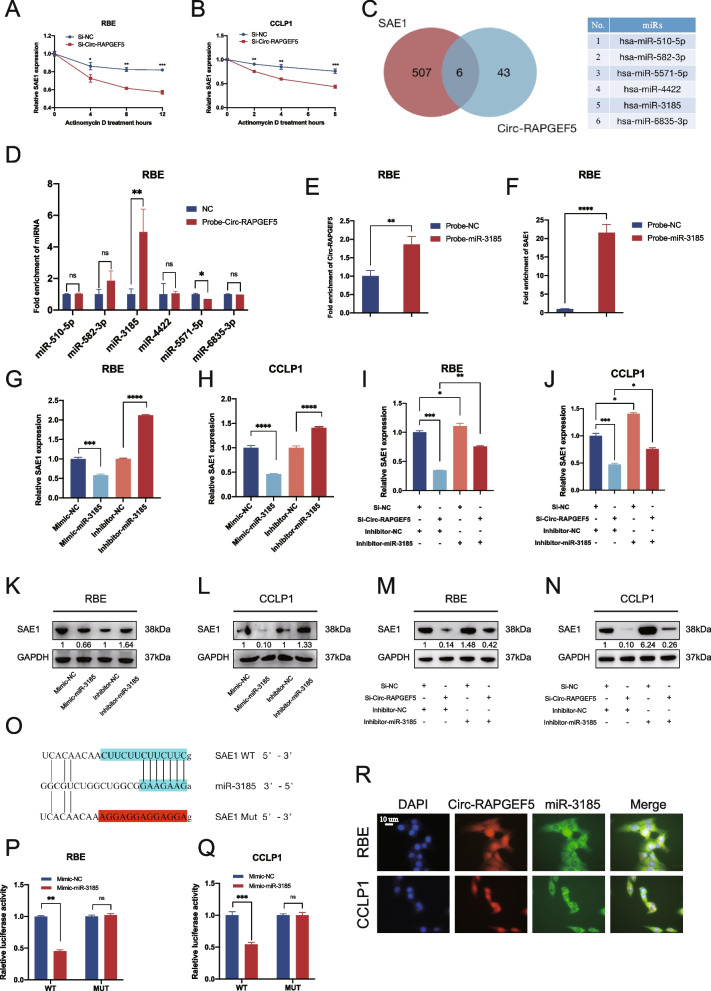
Circ-RAPGEF5 competitively sponges miR-3185 to relieve the degradation of miR-3185 on SAE1. **A-B** qRT-PCR analysis detected relative SAE1 expression in RBE and CCLP1 cells transfected with mock or Si-Circ-RAPGEF5 and treated with Actinomycin D at the indicated time points. **C** Venn plot illustrating the overlap of predicted 49 microRNAs that could be sponged by Circ-RAPGEF5 and predicted 513 microRNAs that potentially bind to the 3' UTR of SAE1. 6 microRNAs were identified. **D** RNA pull-down assay using Circ-RAPGEF5-biotin probe revealed that miR-3185 directly interact with Circ-RAPGEF5 in RBE. **E–F** RNA pull-down assay using a miR-3185-biotin probe confirmed the direct interaction of miR-3185 with Circ-RAPGEF5 (E) and SAE1 (F). **G-H** qRT-PCR analysis testing SAE1 mRNA expression in knockdown and overexpression of miR-3185 in RBE and CCLP1 cells. **I-J** qRT-PCR analysis detecting SAE1 mRNA expression in RBE and CCLP1 cells transfected with Si-NC or Si-Circ-RAPGEF5 and inhibitor-NC or inhibitor-miR-3185. **K-L** Western blot analysis detecting SAE1 protein levels in knockdown and overexpression of miR-3185 of RBE and CCLP1 cells. **M–N** Western blot analysis detecting SAE1 protein levels in RBE and CCLP1 cells transfected with Si-NC or Si-Circ-RAPGEF5 and inhibitor-NC or inhibitor-miR-3185. **O** Schematic illustration showed the alignment of miR-3185 with SAE1 3' UTR (blue) and the mutant nucleotides (red). **P-Q** Dual luciferase reporter assays showed the luciferase activity of wild-type or mutant SAE1 following co-transfection with miR-486-5p mimic or control mimic in RBE and CCLP1 cells. Relative firefly luciferase expression was normalized to that of Renilla luciferase. **R** FISH assays indicated that Circ-RAPGEF5 and miR-3185 co-localized in the cytoplasm. Circ-RPAGEF5 (red), miR-3185 (green), nuclei staining (blue), and merged (yellow) images in RBE and CCLP1 cells. *p < 0.05, **p < 0.01, ***p < 0.001, ****p < 0.0001

The use of qRT-PCR showed that SAE1 mRNA degradation upon actinomycin D treatment to block de novo synthesis was significantly enhanced by Circ-RAPGEF5 downregulation in RBE and CCLP1 cells. This finding suggested that circ-RAPGEF5 enhanced SAE1 mRNA stability (Fig. 7A, B).

Studies have suggested that circular RNAs can act as sponges for miRNAs and regulate gene expression through the ceRNA mechanism [7, 10]. On the basis of our finding that Circ-RAPGEF5 was considerably enriched in the cytoplasm (Fig. 1L-N), we hypothesized that Circ-RAPGEF5 also plays a role in stabilizing SAE1 mRNA through the competing endogenous RNA mechanism. We used Circinteractome (https://circinteractome.nia.nih.gov) and CSCD databases [34] to predict 49 miRNAs that could be sponged by Circ-RAPGEF5. We used the TargetScan database (https://www.targetscan.org) to predict 513 miRNAs that have the potential to bind to the 3' UTR of SAE1. After overlap, we identified six potential miRNA candidates that may play a role in the Circ-RAPGEF5/SAE1 axis (Fig. 7C). The Circ-RAPGEF5 RNA pull-down assay showed that, in RBE cells, only miR-3185 was enriched by Circ-RAPGEF5 (Fig. 7D). Furthermore, the miR-3185 RNA pull-down assay showed that Circ-RAPGEF5 and SAE1 were captured by the miR-3185 biotin probe in RBE cells (Fig. 7E, F). The enrichment efficiency of the Circ-RAPGEF5 biotin probe is shown in Figure S3E. We designed a mimic and inhibitor of miR-3185 and determined its overexpression efficiency in ICC cells (Figure S1K, L). Western blot analysis and qRT-PCR showed that miR-3185 overexpression significantly downregulated SAE1 mRNA and protein levels in ICC cells. In contrast, silencing of miR-3185 significantly increased SAE1 mRNA and protein levels (Fig. 7G, H, K, and L). Furthermore, inhibition of miR-3185 rescued the downregulation of SAE1 caused by the knockdown of Circ-RAPGEF5 (Fig. 7I, J, M, and N). To identify the specific binding region between SAE1 3’ UTR and miR-3185, we constructed miR-3185 binding sites of wild-type and mutant SAE1 3' UTR dual luciferase reporter plasmids (Fig. 7O). Dual luciferase reporter assays showed that wild-type SAE1 luciferase activity was significantly inhibited by miR-3185, whereas mutant SAE1 luciferase activity was unaffected by miR-3185 (Fig. 7P, Q). The FISH assay in RBE and CCLP1 cells further showed that Circ-RAPGEF5 and miR-3185 were co-localized in the cytoplasm of ICC cells (Fig. 7R).

### Circ-RAPGEF5 is involved in SUMOylation and promotes SUMOylation of AKT

**Fig. 8:**
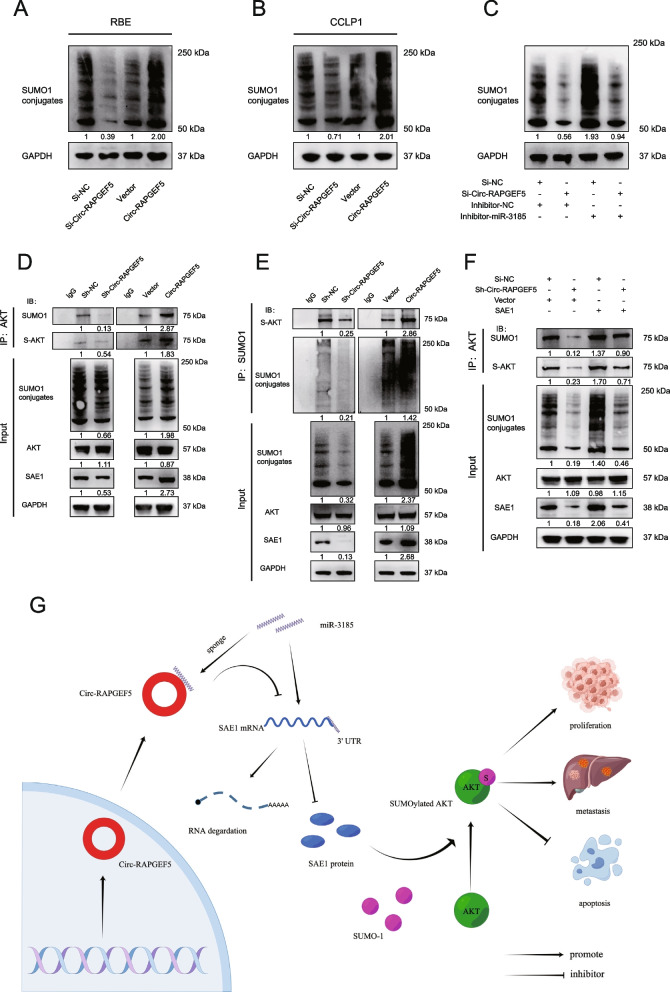
Circ-RAPGEF5 is involved in SUMOylation and promotes the SUMOylation of AKT. **A-B** Western blot analysis accessed the global SUMOylatoin level of Circ-RAPGEF5 silencing and upregulated in RBE and CCLP1 cells. **C** Western blot analysis detecting the global level of SUMOylation in Circ-RAPGEF5 knockdown and overexpression RBE cell while miR-3185 was silenced. **D** CO-IP assays to evaluate AKT SUMOylation level of Circ-RAPGEF5 knockdown and overexpression RBE cell. **E** CO-IP assays to testing SUMO-1 modified AKT level of Circ-RAPGEF5 knockdown and overexpression RBE cell. **F** SUMOylation modification analysis to explore the levels of AKT SUMOylation in Circ-RAPGEF5 knockdown and SAE1 overexpression RBE cell. **G** Hypothetical model for the carcinogenic mechanism of Circ-RAPGEF5. Circ-RAPGEF competitively sponges mir-3185 to stabilize SAE1, thereby promoting SUMOylation and tumor progression

SAE1 is one of the subunits of E1-activating enzymes of SUMOylation, and SAE1 can activate SUMO into its active form to participate in SUMOylation modifications [27]. Therefore, we examined whether Circ-RAPGEF5 is a specific non-coding RNA that can promote SUMOylation levels in ICC cells. Three SUMO proteins, SUMO-1, -2, and -3, are commonly found in humans. qRT-PCR showed that SUMO-1 levels were significantly higher than SUMO-2 and -3 levels in RBE and CCLP1 cells (Figure S3F, G). Therefore, we chose to investigate SUMO-1. Western blot showed that the ability of SUMO-1 to modify the protein was significantly reduced after Circ-RAPGEF5 knockdown (Fig. 8A, B). In contrast, conjugated SUMO-1, which suggested enhanced cell global SUMOylation, was significantly increased after Circ-RAPGEF5 overexpression. Furthermore, the impaired effects of global SUMOylation due to Circ-RAPGEF5 knockdown were rescued by the inhibitor miR-3185 (Fig. 8C).

AKT SUMOylation has been reported to promote tumor proliferation and tumorigenesis [35]. Therefore, we analyzed the relationship between Circ-RAPGEF5, SAE1 expression, and AKT SUMOylation in RBE cells. We used lentiviral infection to establish stably transfected sh-Circ-RAPEGEF5 and Circ-RAPGEF5 overexpression in RBE cells (Figure S1M, N). The immunoprecipitation assay with AKT antibody showed that AKT SUMOylation levels were reduced in Circ-RAPGEF5 knockdown cells. In contrast, AKT SUMOylation levels were significantly increased in Circ-RAPGEF5 overexpressed cells. SUMO-1 was then used to enrich all proteins that could be modified. We found that SUMO-1-modified AKT levels were significantly decreased after Circ-RAPGEF5 knockdown (Fig. 8E). Finally, we found that the diminished effect of AKT SUMOylation caused by Circ-RAPGEF5 downregulation was alleviated by SAE1 overexpression (Fig. 8F). Together, these results suggested that Circ-RAPGEF5 can increase AKT SUMOylation through regulating SAE1 in ICC.

## Discussion

Circ-RAPGEF5 (has_circ_0001681) is a non-coding circular RNA that forms through the back-splicing of five exons (exons 2–6) of the RAPGEF5 gene located on chromosome 7. Previous research has demonstrated that Circ-RAPGEF5 plays various roles in different types of cancers. For instance, Circ-RAPGEF5 acts as a tumor promoter in papillary thyroid carcinoma by competitively binding miR-198, which represses FGFR1 [36]. Another study showed that Circ-RAPGEF5 was lowly expressed in renal cell carcinoma and exerted cancer-inhibiting effects in RCC via the miR-27a-3p/TXNIP pathway [37]. The different effects of Circ-RAPGEF5 may be due to its tissue specificity. The various functions of Circ-RAPGEF5 across different tumors may be due to its tissue specificity (Figure S3A). The mechanism of Circ-RAPGEF5 in the development of ICC is currently unclear. In our study, we analyzed 19 pairs of clinical samples of tumors and adjacent tissues and found that Circ-RAPGEF5 is highly expressed in ICC tumor tissues, suggesting a potential oncogenic role of Circ-RAPGEF5.

Compared with previous studies on Circ-RAPGEF5 [36, 37], we conducted a more comprehensive verification of the loop structure characteristics of Circ-RAPGEF5. We found that Circ-RAPGEF5 is distributed in both the cytoplasm and nucleus of ICC cells and has a longer half-life and stronger resistance to RNase R compared with linear mRNA of RAPGE5 and GAPDH. Additionally, the study suggests that the junction site of Circ-RAPGEF5 is only formed after mRNA splicing, as confirmed by PCR using divergent and convergent primers. Circ-RAPGEF5 depletion significantly attenuated the proliferation, cell cycle, migration, and invasion of ICC cell lines and promoted apoptosis, whereas overexpression of CircRAPGEF5 had the opposite effect. Mechanistically, we found that CircRAPGEF5 competitively binds mir-3185, thereby counteracting its inhibitory effect on SAE1 expression. In ICC, we confirmed the correlation between Circ-RAPGEF4 and SAE1 and revealed that both high expressions of these two genes have a negative impact on survival prognosis. SAE1, an E1-activating enzyme of SUMOylation, could effectively enhance the SUMOylation modification [31]. We have discovered that Circ-RAPGEF5, which regulates the expression of SAE1, can also promote SUMOylation of proteins in ICC cells. We also demonstrated that Circ-RAPGEF5 is effective in the process of AKT SUMOylation, which has been reported to be strongly linked to tumor progression [35, 38]. As far as we know, this study is the first report to establish the significance of SAE1 and SUMOylation in the progression of ICC. And it is a novel mechanism that Circular RNA regulates SAE1 through microRNA. Moreover, we report a carcinogenic circular RNA capable of mediating SUMOylation in ICC, which has not been previously identified (Fig. 8G).

There is growing evidence that Circular RNA plays a crucial role in the development of ICC. Circular RNAs have been found to act as pro-oncogenic factors that facilitate the progression of ICC. For incidence, Circular RNA circACTN4 upregulates YAP1 expression by sponging miR-424-5p, which recruits YBX1 to stimulate FZD7 transcription, thereby promoting ICC proliferation and metastasis [21]. Xu et al. found that circHMGCS1-016 could disrupt the function of T cells by sponging for miR-1236-3p to regulate the expression of CD73 and GAL-8 in ICC [24], suggesting the function of Circular RNA in the tumor immune microenvironment. In addition, the exosomal circular RNA circ-PTPN22 and circ-ADAMTS6 were also identified as characteristic circular RNAs for T cell exhaustion and neutrophil extracellular traps [23]. Circular RNAs are also involved in the metabolic reprogramming process of ICC tumors. Yu et al. reported a circular RNA, CircMBOAT2, which binds to PTBP1 and protects PTBP1 from ubiquitin/proteasome-dependent degradation, thereby inhibiting the nuclear export of FASN mRNA, promoting lipid metabolism and regulation of redox homeostasis to facilitate ICC progression [25]. The mechanism of Circular RNAs in the development of ICC is bidirectional. CircNFIB competitively binds MEK1 and induces dissociation between MEK1 and ERK2, resulting in blocked ERK signaling and inhibition of tumor metastasis [22]. The circular RNA promoting SUMOylation reported in the current study is a novel mechanism in regulating the progression of ICC.

SAE1 is one of the key enzymes in the SUMOylation process, and together with another subunit, SAE2, constitutes a SUMO E1 activating enzyme to catalyze the activation of SUMO.

The expression level of SAE1 tends to be altered in a variety of tumors, suggesting its possible involvement in tumorigenesis and progression. For example, several studies have shown that the expression level of SAE1 is increased in some cancer types, such as hepatocellular carcinoma, breast cancer, gastric cancer, pancreatic cancer, and lung cancer [29, 31, 39–41]. Therefore, SAE1 is a protein worth studying, and researching it can help better understand the SUMOylation process as well as the mechanism of tumorigenesis. In particular, in HCC, SAE1 expression is closely correlated with the tumor stage, metastatic phenotype, and survival rate, indicating that SAE1 has excellent diagnostic value in HCC [39]. Functionally, SAE1 promotes the proliferation and invasion of HCC cells and inhibits apoptosis, whereas the absence of SAE1 blocks the tumor cell cycle [30]. Mechanistically, SAE1 promotes the malignant biological behavior of HCC cells through SUMOylation and phosphorylation of the mTOR signaling pathway [30]. SAE1 has also been reported to promote glioma cancer progression through enhanced Akt SUMOylation [38].

However, the function and mechanism of SAE1 in the development of ICC remains unclear. In our study, we first found that high expression of SAE1 was significantly associated with poor prognosis in ICC patients and that SAE1 significantly promoted tumor proliferation and migration. It indicates that SAE1 may be an important potential therapeutic target for ICC. We look forward to future studies investigating the oncogenic effects of inhibitors of SAE1 in vivo and in patients.

Post-translational modifications include phosphorylation/dephosphorylation, acetylation/deacetylation, ubiquitination/deubiquitination, neddylation/deneddylation, and SUMOylation/deSUMOylation to regulate protein activity and expression [42]. SUMOylation is an intracellular post-translational modification that regulates biological functions such as activity, subcellular localization, stability, and interactions of target proteins by covalently attaching small ubiquitin-like proteins (SUMO) to the target protein [43].

In mammals, four distinct isoforms, SUMO-1, -2, -3, and -4, have been identified, whereas SUMO-1, -2, and -3 are usually highly expressed in humans. SUMOylation plays an important role in tumorigenesis and progression. SUMOylation can regulate the function and expression of various tumor-related proteins, including transcription factors, DNA repair factors, apoptosis-related factors, and cell cycle regulators [28]. Many different receptors or intracellular signaling molecules have been shown to be modified by SUMO and thus significantly promote carcinogeneses, such as ICF-IR [44] type I TGF-β receptor [45], and HIF-1α [46]. Regulation of SUMOylation is a novel target for treating cancers, and SUMOylation inhibitors have been reported to be used in preclinical models to treat pancreatic ductal adenocarcinoma [47].

In the present study, since Circ-RAPGEF5 significantly increased the expression of SAE1, we confirmed that Circ-RAPGEF5 promotes the overall SUMOylation of ICC cells. AKT is thought to be one of the driver genes for ICC and HCC [48, 49], and it has been suggested that the process of AKT SUMOylation can regulate the proliferation and invasion of tumor cells [35]. For this reason, we explored whether Circ-RAPGEF5 exerts an oncogenic mechanism by enhancing AKT SUMOylation. The results showed that the depletion of Circ-RAPGEF5 significantly impaired the process of AKT SUMOylation and that this process could be rescued by SAE1, suggesting that Circ-RAPGEF5-mediated SUMOylation plays an important role in tumor formation and progression. It also offers novel insights and potential targets for the treatment of ICC. The regulation of Circ-RAPGEF5 and SAE1 could hold significant therapeutic value in the management, which was confirmed by the slowing of tumor growth due to our injection of Sh-Circ-RAPGEF5 lentiviral solution (Fig. 4E).

## Conclusions

In summary, we uncovered a novel mechanism by which Circular RNAs regulate SUMOylation to promote ICC tumor progression. We demonstrated that Circ-RPAGEF5 upregulation was associated with poor prognosis in ICC patients. We further discovered that Circ-RAPGEF5 competitively binds miR-3185 to stabilize SAE1 from degradation, promote overall AKT SUMOylation, and induce tumor proliferation and migration.

### Supplementary Information

**Additional file 1: Table S1.** Univariate and multivariate COX regression analysis of the 91 ICC patients.** Table S2.** Primers used in this study.** Table S3.** Antibodies and reagents used in this study.** Table S4.** FISH probes used in this study.** Table S5.** Biotinylated probes used in this study.** Figure S1.** The expression levels of Circ-RAPGEF5, SAE1 and miR-3185 in indicated cells. A-B qRT-PCR and western blot analysis detected the expression level of Circ-RAPGEF5 and liner RAPGEF5 in RBE cells after treatment with Si-Circ-RAPGEF5 or Si-NC. C-F The transfected efficiency of Si-Circ-RAPGEF5 and Circ-RAPGEF5 overexpression plasmid in RBE and CCLP1 cells. G-J The transfected efficiency of Si-SAE1 and SAE1 overexpression plasmid in RBE and CCLP1 cells. K-L the overexpression efficiency of miR-3184 mimic in RBE and CCLP1cells. M-N qRT-PCR analysis detected the Circ-RAPGEF5 expression of stably transfected Sh-Circ-RAPGEF5 and Circ-RAPGEF5 RBE cells. *p < 0.05, **p < 0.01, ***p < 0.001, ****p < 0.0001.** Figure S2.** Circ-RAPGEF5 inhibits apoptosis and promotes migration in ICC cells. A-B Cell apoptosis analysis detected by flow cytometry in Circ-RAPGEF5 knockdown or overexpression cells. C-D The migration ability was assessed by transwell assay in Circ-RAPGEF5 knockdown or overexpression cells. All data are presented as the means ± SD of three independent experiments. *p < 0.05, **p < 0.01, ***p < 0.001, ****p < 0.0001.** Figure S3.** A Differential expression of SAE1 ICC tumor tissues and adjacent normal tissues in TCGA data. B Kaplan-Meier survival curves of external sequencing data from Dong et al. C qRT-PCR analysis of the relative expression levels of SAE1 in xenografts tissue of groups treated with Sh-Circ-RAPGEF5 and Sh-NC. D Representative IHC images for SAE1 of Sh-NC and Sh-Circ-RAPGEF5 virus treated patient-derived tumor xenograft. E qRT-PCR verified the enrichment efficiency of the Circ-RAPGEF5-biotin probe. F-G qRT-PCR detecting relative SUMO expression in RBE and CCLP1 cells. *p < 0.05, **p < 0.01, ***p < 0.001, ****p < 0.0001.** Figure S4.** A-B The remaining two Western blot experiments accessed SAE1 protein level after changing the expression of Circ-RAPGEF5 in RBE cell. C-D The remaining two Western blot experiments accessed SAE1 protein level after changing the expression of Circ-RAPGEF5 in CCLP1 cell.** Figure S5.** A-B The remaining two Western blot experiments detecting SAE1 protein level in knockdown and overexpression of miR-3185 of RBE cell. C-D The remaining two Western blot experiments detecting SAE1 protein level in knockdown and overexpression of miR-3185 of CCLP1 cell. E-F The remaining two Western blot experiments detecting SAE1 protein level in RBE cell transfected with Si-NC or Si-Circ-RAPGEF5 and inhibitor-NC or inhibitor-miR-3185. G-H The remaining two Western blot experiments detecting SAE1 protein level in CCLP1 cell transfected with Si-NC or Si-Circ-RAPGEF5 and inhibitor-NC or inhibitor-miR-3185.** Figure S6.** A-B The remaining two Western blot experiments accessed the global SUMOylatoin level of Circ-RAPGEF5 silencing and upregulated in RBE cells. C-D The remaining two Western blot experiments accessed the global SUMOylatoin level of Circ-RAPGEF5 silencing and upregulated in CCLP1 cells. E-F The remaining two Western blot experiments detecting the global level of SUMOylation in Circ-RAPGEF5 knockdown and overexpression RBE cell while miR-3185 was silenced.** Figure S7.** A-B The remaining two Western blot experiments of CO-IP assays to evaluate AKT SUMOylation level of Circ-RAPGEF5 knockdown and overexpression RBE cell. C-D The remaining two Western blot experiments of CO-IP assays to testing SUMO-1 modified AKT level of Circ-RAPGEF5 knockdown and overexpression RBE cell. E-F The remaining two Western blot experiments of SUMOylation modification analysis to explore the levels of AKT SUMOylation in Circ-RAPGEF5 knockdown and SAE1 overexpression RBE cell.** Figure S8.** A-B The remaining two Western blot experiments detected the expression level of liner RAPGEF5 in RBE cells after treatment with Si-Circ-RAPGEF5 or Si-NC.

## Data Availability

The data supporting the findings of this article is included within this article and its additional files.

## Abbreviations

ICC: Intrahepatic cholangiocarcinoma
HCC: Hepatocellular carcinoma
SAE1: SUMO-activating enzyme subunit 1
ceRNAs: Competing endogenous RNAs
miRNA: MicroRNA
IHC: Immunohistochemical
SiRNAs: Small interfering RNA
ShRNAs: Short hairpin RNAs
qRT-PCR: Quantitative real-time Polymerase Chain Reaction
CDX: Cell-derived xenograft
PDX: Patient-derived xenograft
FISH: Fluorescence in situ hybridization
Co-IP: Coimmunoprecipitation
TCGA: The Cancer Genome Atlas
3′-UTR: 3′-Untranslated region
SUMO: Small ubiquitin-related modifier
AKT: RAC-alpha serine/threonine-protein kinase
