# Supplementary figures and images for: Circ-RAPGEF5 promotes intrahepatic cholangiocarcinoma progression by stabilizing SAE1 to facilitate SUMOylation
Source: J Exp Clin Cancer Res. 2023 Sep 13;42:239. doi: 10.1186/s13046-023-02813-y (PMC10498551; doi:10.1186/s13046-023-02813-y)

A

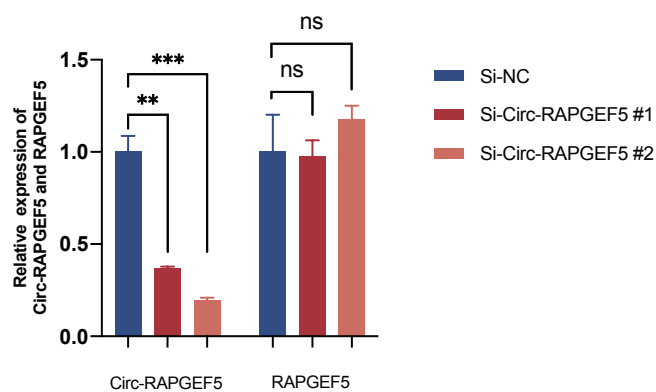

B

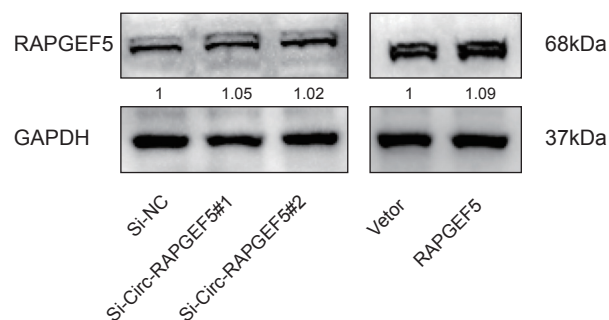

C

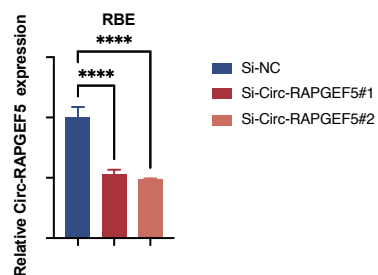

D

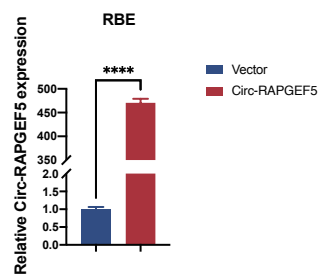

E

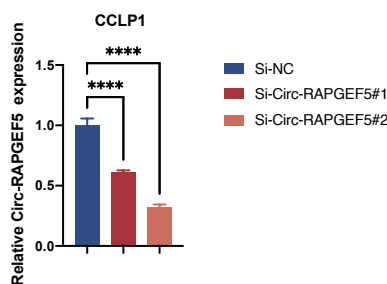

F

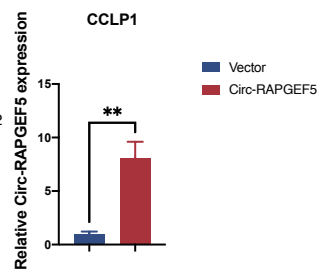

G

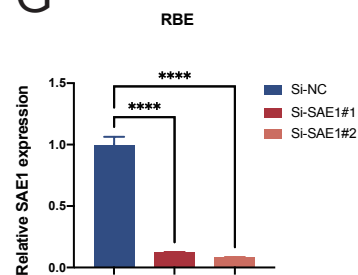

H

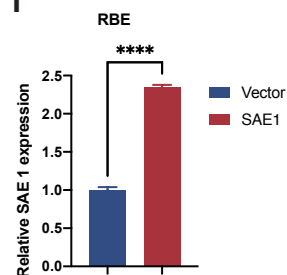

I

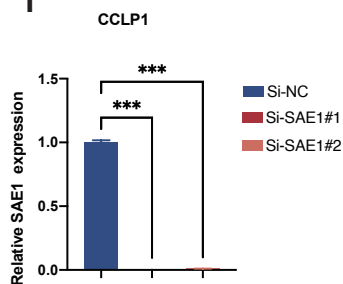

J

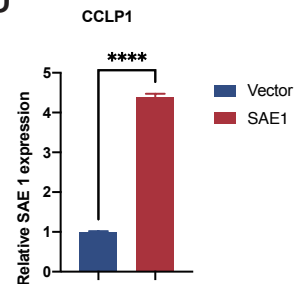

K

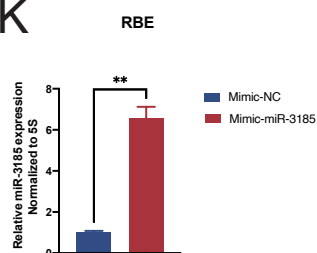

L

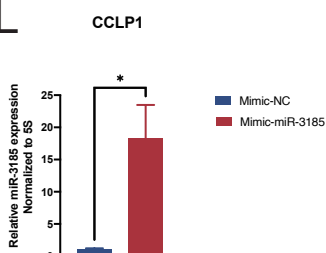

M

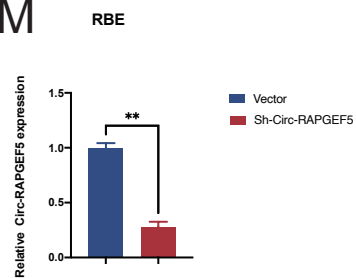

N

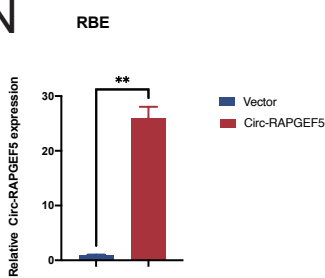

Supplement: Supplementary file 1 — Additional file 1: Table S1. Univariate and multivariate COX regression analysis of the 91 ICC patients. Table S2. Primers used in this study. Table S3. Antibodies and reagents used in this study. Table S4. FISH probes used in this study. Table S5. Biotinylated probes used in this study. Figure S1. The expression levels of Circ-RAPGEF5, SAE1 and miR-3185 in indicated cells. A-B qRT-PCR and western blot analysis detected the expression level of Circ-RAPGEF5 and liner RAPGEF5 in RBE cells after treatment with Si-Circ-RAPGEF5 or Si-NC. C-F The transfected efficiency of Si-Circ-RAPGEF5 and Circ-RAPGEF5 overexpression plasmid in RBE and CCLP1 cells. G-J The transfected efficiency of Si-SAE1 and SAE1 overexpression plasmid in RBE and CCLP1 cells. K-L the overexpression efficiency of miR-3184 mimic in RBE and CCLP1cells. M-N qRT-PCR analysis detected the Circ-RAPGEF5 expression of stably transfected Sh-Circ-RAPGEF5 and Circ-RAPGEF5 RBE cells. *p < 0.05, **p < 0.01, ***p < 0.001, ****p < 0.0001. Figure S2. Circ-RAPGEF5 inhibits apoptosis and promotes migration in ICC cells. A-B Cell apoptosis analysis detected by flow cytometry in Circ-RAPGEF5 knockdown or overexpression cells. C-D The migration ability was assessed by transwell assay in Circ-RAPGEF5 knockdown or overexpression cells. All data are presented as the means ± SD of three independent experiments. *p < 0.05, **p < 0.01, ***p < 0.001, ****p < 0.0001. Figure S3. A Differential expression of SAE1 ICC tumor tissues and adjacent normal tissues in TCGA data. B Kaplan-Meier survival curves of external sequencing data from Dong et al. C qRT-PCR analysis of the relative expression levels of SAE1 in xenografts tissue of groups treated with Sh-Circ-RAPGEF5 and Sh-NC. D Representative IHC images for SAE1 of Sh-NC and Sh-Circ-RAPGEF5 virus treated patient-derived tumor xenograft. E qRT-PCR verified the enrichment efficiency of the Circ-RAPGEF5-biotin probe. F-G qRT-PCR detecting relative SUMO expression in RBE and CCLP1 cells [file 13046_2023_2813_MOESM1_ESM.zip › ADDITIONAL FILE 1/Figure S1.pdf]

A

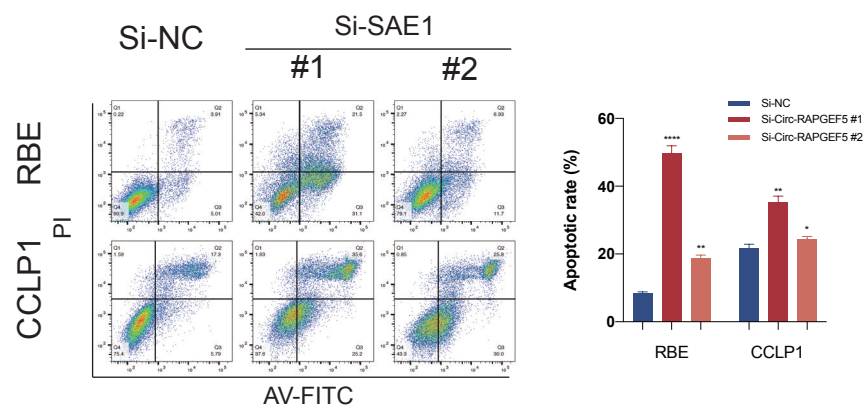

B

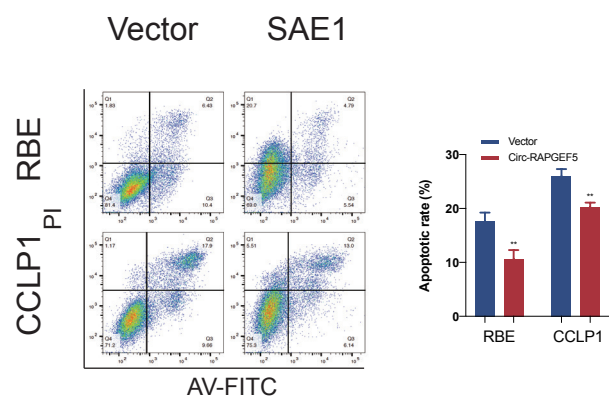

C

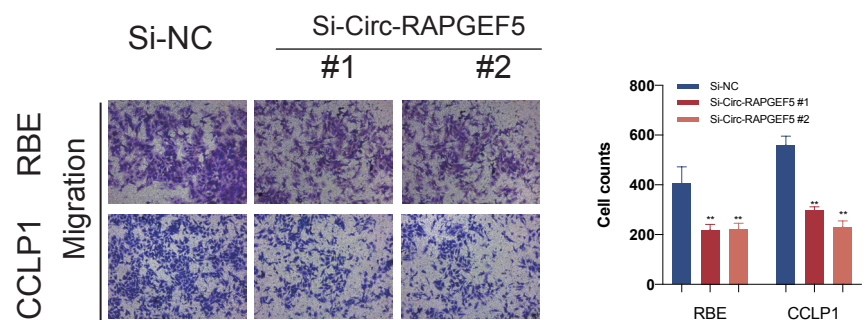

D

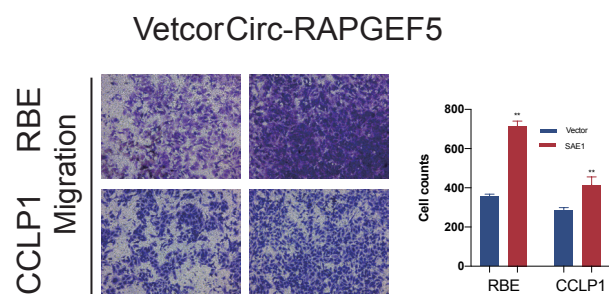

Supplement: Supplementary file 1 — Additional file 1: Table S1. Univariate and multivariate COX regression analysis of the 91 ICC patients. Table S2. Primers used in this study. Table S3. Antibodies and reagents used in this study. Table S4. FISH probes used in this study. Table S5. Biotinylated probes used in this study. Figure S1. The expression levels of Circ-RAPGEF5, SAE1 and miR-3185 in indicated cells. A-B qRT-PCR and western blot analysis detected the expression level of Circ-RAPGEF5 and liner RAPGEF5 in RBE cells after treatment with Si-Circ-RAPGEF5 or Si-NC. C-F The transfected efficiency of Si-Circ-RAPGEF5 and Circ-RAPGEF5 overexpression plasmid in RBE and CCLP1 cells. G-J The transfected efficiency of Si-SAE1 and SAE1 overexpression plasmid in RBE and CCLP1 cells. K-L the overexpression efficiency of miR-3184 mimic in RBE and CCLP1cells. M-N qRT-PCR analysis detected the Circ-RAPGEF5 expression of stably transfected Sh-Circ-RAPGEF5 and Circ-RAPGEF5 RBE cells. *p < 0.05, **p < 0.01, ***p < 0.001, ****p < 0.0001. Figure S2. Circ-RAPGEF5 inhibits apoptosis and promotes migration in ICC cells. A-B Cell apoptosis analysis detected by flow cytometry in Circ-RAPGEF5 knockdown or overexpression cells. C-D The migration ability was assessed by transwell assay in Circ-RAPGEF5 knockdown or overexpression cells. All data are presented as the means ± SD of three independent experiments. *p < 0.05, **p < 0.01, ***p < 0.001, ****p < 0.0001. Figure S3. A Differential expression of SAE1 ICC tumor tissues and adjacent normal tissues in TCGA data. B Kaplan-Meier survival curves of external sequencing data from Dong et al. C qRT-PCR analysis of the relative expression levels of SAE1 in xenografts tissue of groups treated with Sh-Circ-RAPGEF5 and Sh-NC. D Representative IHC images for SAE1 of Sh-NC and Sh-Circ-RAPGEF5 virus treated patient-derived tumor xenograft. E qRT-PCR verified the enrichment efficiency of the Circ-RAPGEF5-biotin probe. F-G qRT-PCR detecting relative SUMO expression in RBE and CCLP1 cells [file 13046_2023_2813_MOESM1_ESM.zip › ADDITIONAL FILE 1/Figure S2.pdf]

A

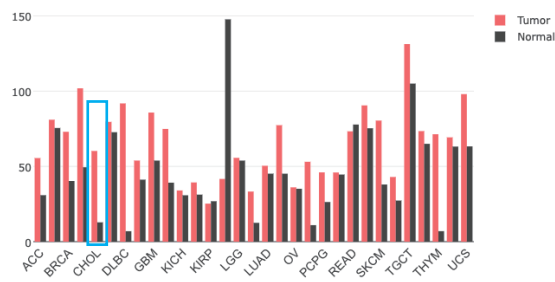

B

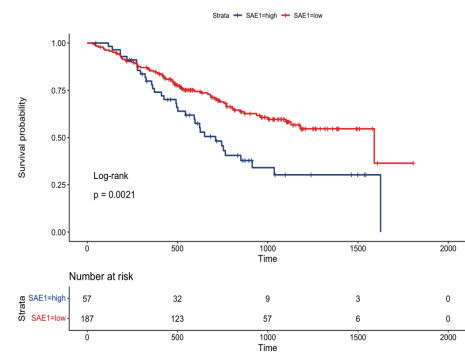

C

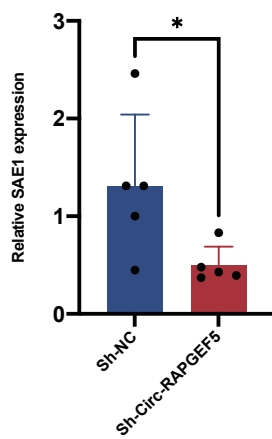

D

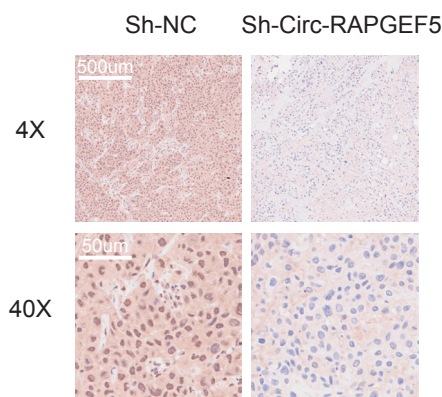

E

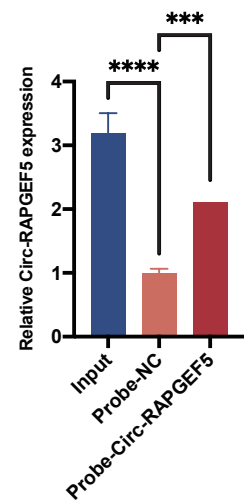

F

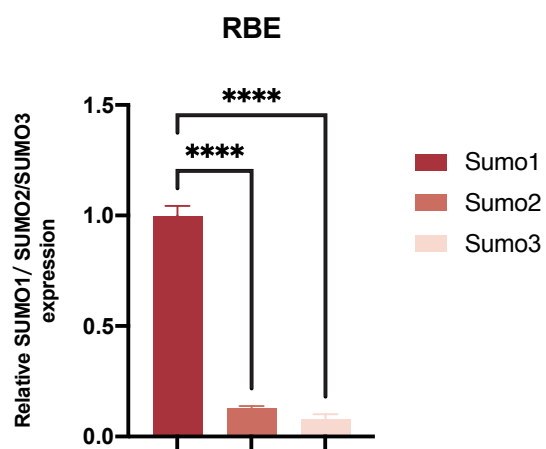

G

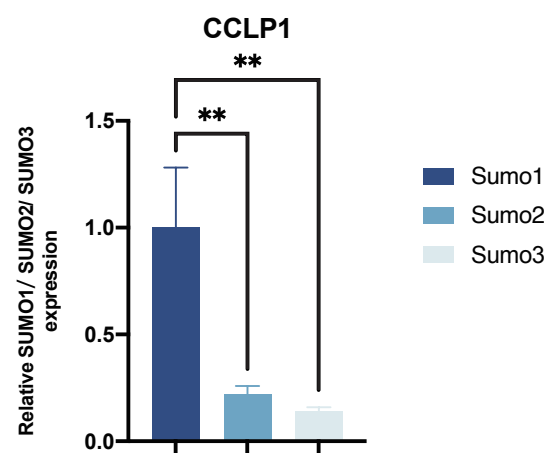

Supplement: Supplementary file 1 — Additional file 1: Table S1. Univariate and multivariate COX regression analysis of the 91 ICC patients. Table S2. Primers used in this study. Table S3. Antibodies and reagents used in this study. Table S4. FISH probes used in this study. Table S5. Biotinylated probes used in this study. Figure S1. The expression levels of Circ-RAPGEF5, SAE1 and miR-3185 in indicated cells. A-B qRT-PCR and western blot analysis detected the expression level of Circ-RAPGEF5 and liner RAPGEF5 in RBE cells after treatment with Si-Circ-RAPGEF5 or Si-NC. C-F The transfected efficiency of Si-Circ-RAPGEF5 and Circ-RAPGEF5 overexpression plasmid in RBE and CCLP1 cells. G-J The transfected efficiency of Si-SAE1 and SAE1 overexpression plasmid in RBE and CCLP1 cells. K-L the overexpression efficiency of miR-3184 mimic in RBE and CCLP1cells. M-N qRT-PCR analysis detected the Circ-RAPGEF5 expression of stably transfected Sh-Circ-RAPGEF5 and Circ-RAPGEF5 RBE cells. *p < 0.05, **p < 0.01, ***p < 0.001, ****p < 0.0001. Figure S2. Circ-RAPGEF5 inhibits apoptosis and promotes migration in ICC cells. A-B Cell apoptosis analysis detected by flow cytometry in Circ-RAPGEF5 knockdown or overexpression cells. C-D The migration ability was assessed by transwell assay in Circ-RAPGEF5 knockdown or overexpression cells. All data are presented as the means ± SD of three independent experiments. *p < 0.05, **p < 0.01, ***p < 0.001, ****p < 0.0001. Figure S3. A Differential expression of SAE1 ICC tumor tissues and adjacent normal tissues in TCGA data. B Kaplan-Meier survival curves of external sequencing data from Dong et al. C qRT-PCR analysis of the relative expression levels of SAE1 in xenografts tissue of groups treated with Sh-Circ-RAPGEF5 and Sh-NC. D Representative IHC images for SAE1 of Sh-NC and Sh-Circ-RAPGEF5 virus treated patient-derived tumor xenograft. E qRT-PCR verified the enrichment efficiency of the Circ-RAPGEF5-biotin probe. F-G qRT-PCR detecting relative SUMO expression in RBE and CCLP1 cells [file 13046_2023_2813_MOESM1_ESM.zip › ADDITIONAL FILE 1/Figure S3.pdf]

**A**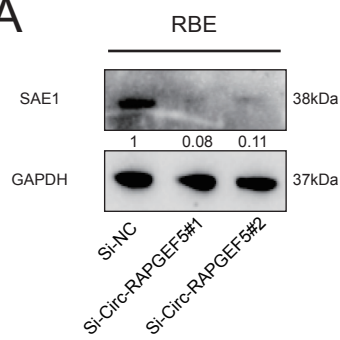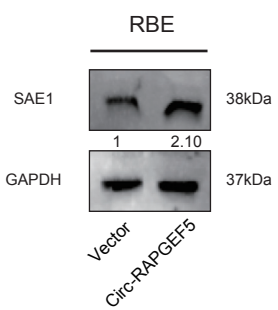**C**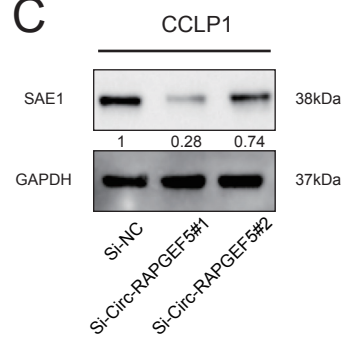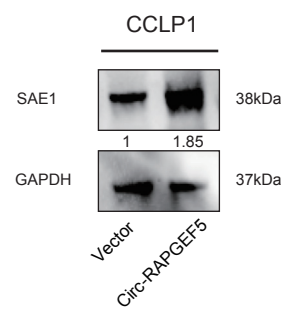**B**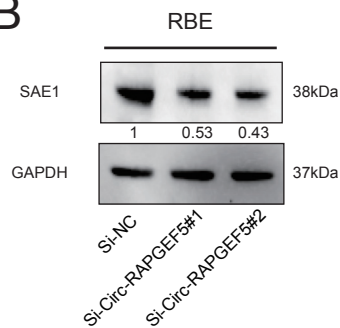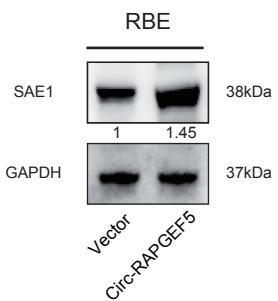**D**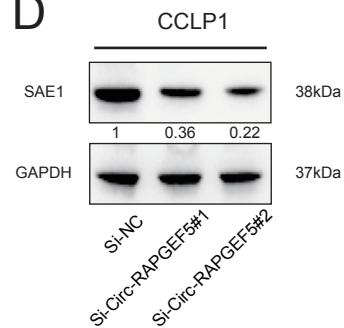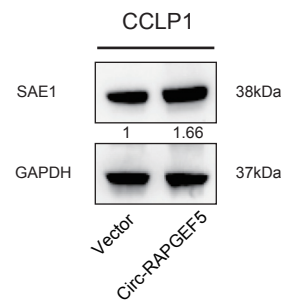

Supplement: Supplementary file 1 — Additional file 1: Table S1. Univariate and multivariate COX regression analysis of the 91 ICC patients. Table S2. Primers used in this study. Table S3. Antibodies and reagents used in this study. Table S4. FISH probes used in this study. Table S5. Biotinylated probes used in this study. Figure S1. The expression levels of Circ-RAPGEF5, SAE1 and miR-3185 in indicated cells. A-B qRT-PCR and western blot analysis detected the expression level of Circ-RAPGEF5 and liner RAPGEF5 in RBE cells after treatment with Si-Circ-RAPGEF5 or Si-NC. C-F The transfected efficiency of Si-Circ-RAPGEF5 and Circ-RAPGEF5 overexpression plasmid in RBE and CCLP1 cells. G-J The transfected efficiency of Si-SAE1 and SAE1 overexpression plasmid in RBE and CCLP1 cells. K-L the overexpression efficiency of miR-3184 mimic in RBE and CCLP1cells. M-N qRT-PCR analysis detected the Circ-RAPGEF5 expression of stably transfected Sh-Circ-RAPGEF5 and Circ-RAPGEF5 RBE cells. *p < 0.05, **p < 0.01, ***p < 0.001, ****p < 0.0001. Figure S2. Circ-RAPGEF5 inhibits apoptosis and promotes migration in ICC cells. A-B Cell apoptosis analysis detected by flow cytometry in Circ-RAPGEF5 knockdown or overexpression cells. C-D The migration ability was assessed by transwell assay in Circ-RAPGEF5 knockdown or overexpression cells. All data are presented as the means ± SD of three independent experiments. *p < 0.05, **p < 0.01, ***p < 0.001, ****p < 0.0001. Figure S3. A Differential expression of SAE1 ICC tumor tissues and adjacent normal tissues in TCGA data. B Kaplan-Meier survival curves of external sequencing data from Dong et al. C qRT-PCR analysis of the relative expression levels of SAE1 in xenografts tissue of groups treated with Sh-Circ-RAPGEF5 and Sh-NC. D Representative IHC images for SAE1 of Sh-NC and Sh-Circ-RAPGEF5 virus treated patient-derived tumor xenograft. E qRT-PCR verified the enrichment efficiency of the Circ-RAPGEF5-biotin probe. F-G qRT-PCR detecting relative SUMO expression in RBE and CCLP1 cells [file 13046_2023_2813_MOESM1_ESM.zip › ADDITIONAL FILE 1/Figure S4.pdf]

A

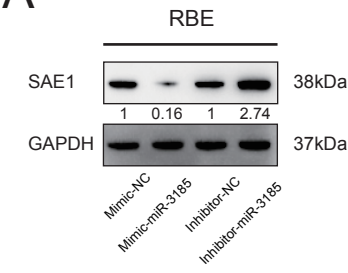

C

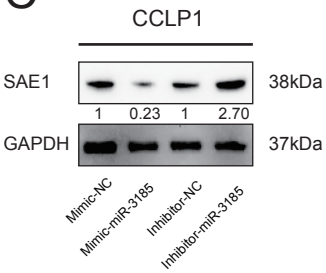

E

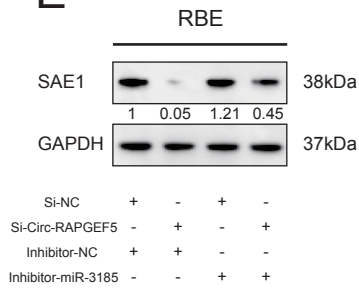

G

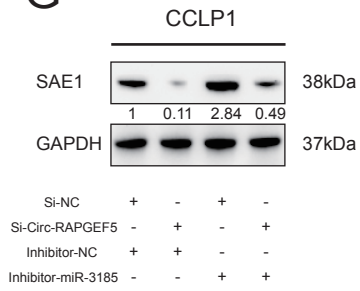

B

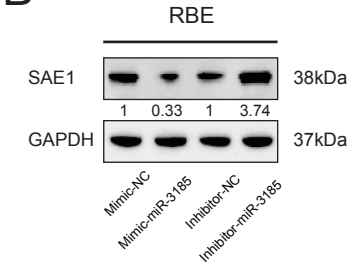

D

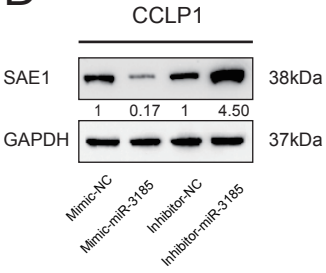

F

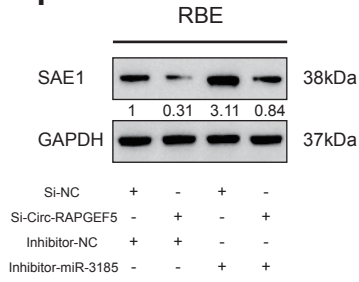

H

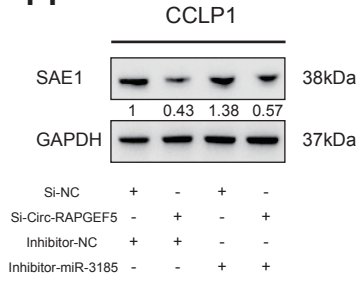

Supplement: Supplementary file 1 — Additional file 1: Table S1. Univariate and multivariate COX regression analysis of the 91 ICC patients. Table S2. Primers used in this study. Table S3. Antibodies and reagents used in this study. Table S4. FISH probes used in this study. Table S5. Biotinylated probes used in this study. Figure S1. The expression levels of Circ-RAPGEF5, SAE1 and miR-3185 in indicated cells. A-B qRT-PCR and western blot analysis detected the expression level of Circ-RAPGEF5 and liner RAPGEF5 in RBE cells after treatment with Si-Circ-RAPGEF5 or Si-NC. C-F The transfected efficiency of Si-Circ-RAPGEF5 and Circ-RAPGEF5 overexpression plasmid in RBE and CCLP1 cells. G-J The transfected efficiency of Si-SAE1 and SAE1 overexpression plasmid in RBE and CCLP1 cells. K-L the overexpression efficiency of miR-3184 mimic in RBE and CCLP1cells. M-N qRT-PCR analysis detected the Circ-RAPGEF5 expression of stably transfected Sh-Circ-RAPGEF5 and Circ-RAPGEF5 RBE cells. *p < 0.05, **p < 0.01, ***p < 0.001, ****p < 0.0001. Figure S2. Circ-RAPGEF5 inhibits apoptosis and promotes migration in ICC cells. A-B Cell apoptosis analysis detected by flow cytometry in Circ-RAPGEF5 knockdown or overexpression cells. C-D The migration ability was assessed by transwell assay in Circ-RAPGEF5 knockdown or overexpression cells. All data are presented as the means ± SD of three independent experiments. *p < 0.05, **p < 0.01, ***p < 0.001, ****p < 0.0001. Figure S3. A Differential expression of SAE1 ICC tumor tissues and adjacent normal tissues in TCGA data. B Kaplan-Meier survival curves of external sequencing data from Dong et al. C qRT-PCR analysis of the relative expression levels of SAE1 in xenografts tissue of groups treated with Sh-Circ-RAPGEF5 and Sh-NC. D Representative IHC images for SAE1 of Sh-NC and Sh-Circ-RAPGEF5 virus treated patient-derived tumor xenograft. E qRT-PCR verified the enrichment efficiency of the Circ-RAPGEF5-biotin probe. F-G qRT-PCR detecting relative SUMO expression in RBE and CCLP1 cells [file 13046_2023_2813_MOESM1_ESM.zip › ADDITIONAL FILE 1/Figure S5.pdf]

A

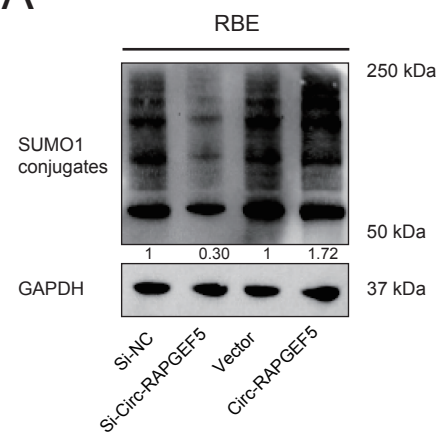

B

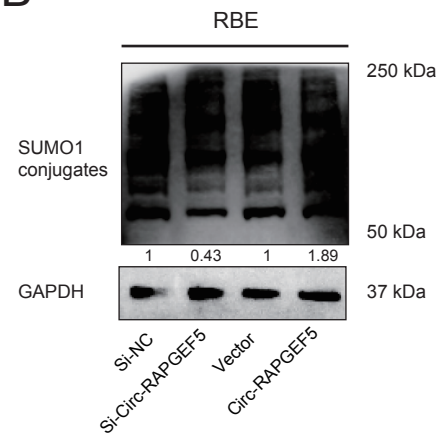

C

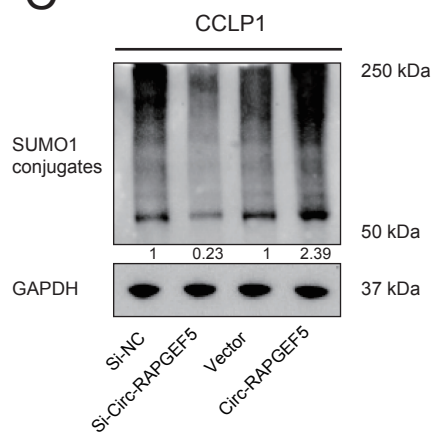

D

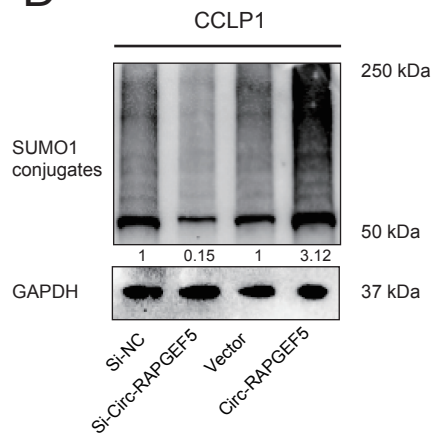

E

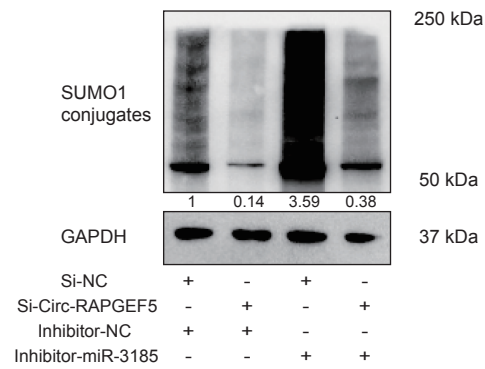

F

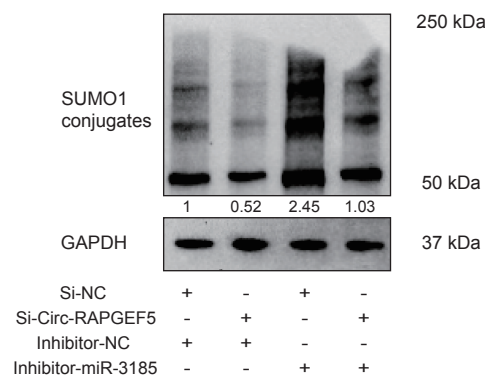

Supplement: Supplementary file 1 — Additional file 1: Table S1. Univariate and multivariate COX regression analysis of the 91 ICC patients. Table S2. Primers used in this study. Table S3. Antibodies and reagents used in this study. Table S4. FISH probes used in this study. Table S5. Biotinylated probes used in this study. Figure S1. The expression levels of Circ-RAPGEF5, SAE1 and miR-3185 in indicated cells. A-B qRT-PCR and western blot analysis detected the expression level of Circ-RAPGEF5 and liner RAPGEF5 in RBE cells after treatment with Si-Circ-RAPGEF5 or Si-NC. C-F The transfected efficiency of Si-Circ-RAPGEF5 and Circ-RAPGEF5 overexpression plasmid in RBE and CCLP1 cells. G-J The transfected efficiency of Si-SAE1 and SAE1 overexpression plasmid in RBE and CCLP1 cells. K-L the overexpression efficiency of miR-3184 mimic in RBE and CCLP1cells. M-N qRT-PCR analysis detected the Circ-RAPGEF5 expression of stably transfected Sh-Circ-RAPGEF5 and Circ-RAPGEF5 RBE cells. *p < 0.05, **p < 0.01, ***p < 0.001, ****p < 0.0001. Figure S2. Circ-RAPGEF5 inhibits apoptosis and promotes migration in ICC cells. A-B Cell apoptosis analysis detected by flow cytometry in Circ-RAPGEF5 knockdown or overexpression cells. C-D The migration ability was assessed by transwell assay in Circ-RAPGEF5 knockdown or overexpression cells. All data are presented as the means ± SD of three independent experiments. *p < 0.05, **p < 0.01, ***p < 0.001, ****p < 0.0001. Figure S3. A Differential expression of SAE1 ICC tumor tissues and adjacent normal tissues in TCGA data. B Kaplan-Meier survival curves of external sequencing data from Dong et al. C qRT-PCR analysis of the relative expression levels of SAE1 in xenografts tissue of groups treated with Sh-Circ-RAPGEF5 and Sh-NC. D Representative IHC images for SAE1 of Sh-NC and Sh-Circ-RAPGEF5 virus treated patient-derived tumor xenograft. E qRT-PCR verified the enrichment efficiency of the Circ-RAPGEF5-biotin probe. F-G qRT-PCR detecting relative SUMO expression in RBE and CCLP1 cells [file 13046_2023_2813_MOESM1_ESM.zip › ADDITIONAL FILE 1/Figure S6.pdf]

A

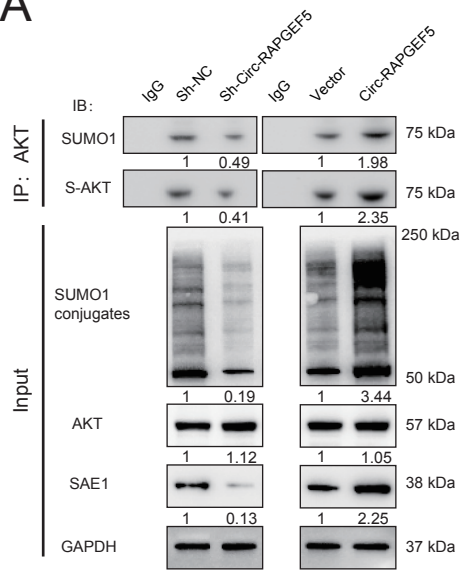

B

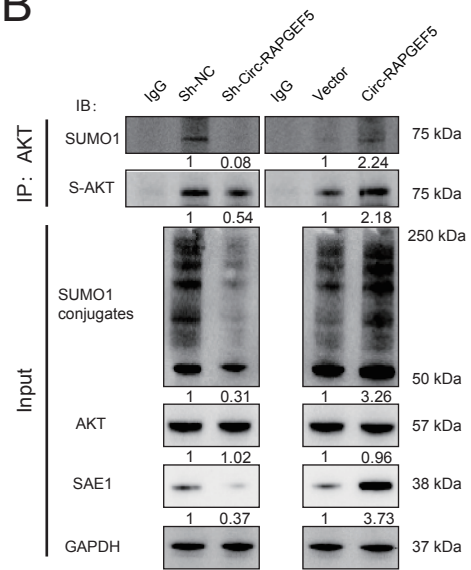

C

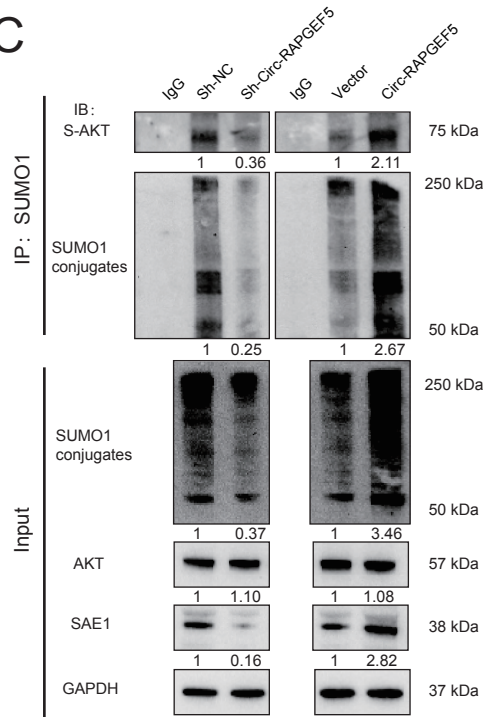

D

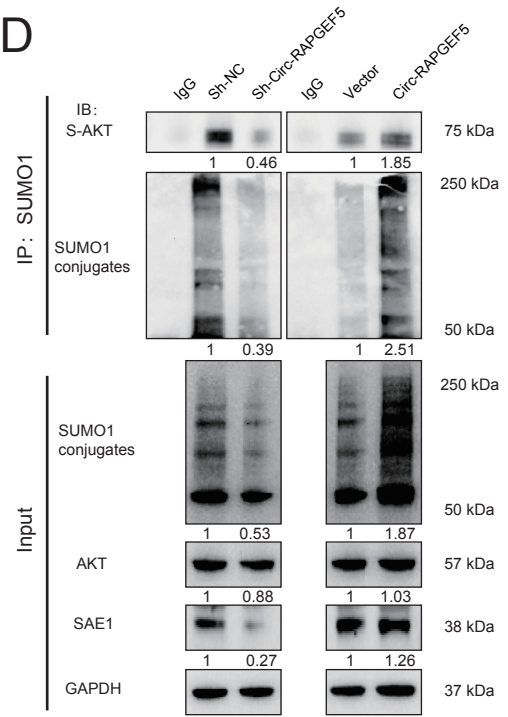

E

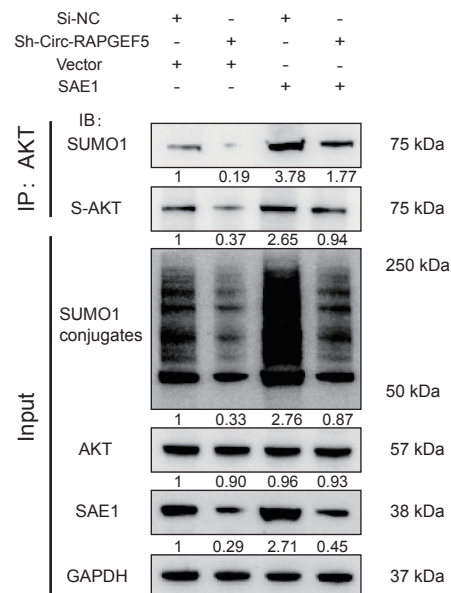

F

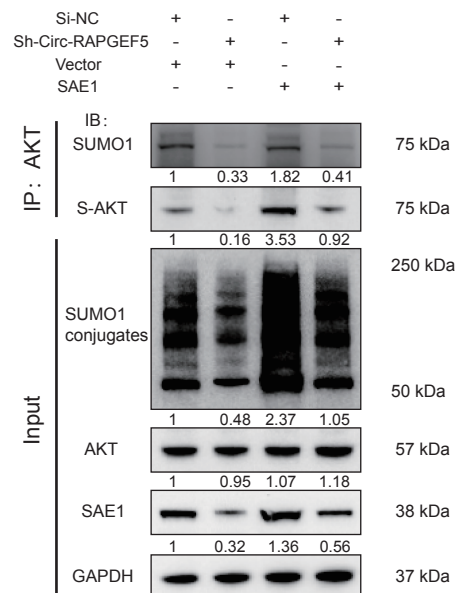

Supplement: Supplementary file 1 — Additional file 1: Table S1. Univariate and multivariate COX regression analysis of the 91 ICC patients. Table S2. Primers used in this study. Table S3. Antibodies and reagents used in this study. Table S4. FISH probes used in this study. Table S5. Biotinylated probes used in this study. Figure S1. The expression levels of Circ-RAPGEF5, SAE1 and miR-3185 in indicated cells. A-B qRT-PCR and western blot analysis detected the expression level of Circ-RAPGEF5 and liner RAPGEF5 in RBE cells after treatment with Si-Circ-RAPGEF5 or Si-NC. C-F The transfected efficiency of Si-Circ-RAPGEF5 and Circ-RAPGEF5 overexpression plasmid in RBE and CCLP1 cells. G-J The transfected efficiency of Si-SAE1 and SAE1 overexpression plasmid in RBE and CCLP1 cells. K-L the overexpression efficiency of miR-3184 mimic in RBE and CCLP1cells. M-N qRT-PCR analysis detected the Circ-RAPGEF5 expression of stably transfected Sh-Circ-RAPGEF5 and Circ-RAPGEF5 RBE cells. *p < 0.05, **p < 0.01, ***p < 0.001, ****p < 0.0001. Figure S2. Circ-RAPGEF5 inhibits apoptosis and promotes migration in ICC cells. A-B Cell apoptosis analysis detected by flow cytometry in Circ-RAPGEF5 knockdown or overexpression cells. C-D The migration ability was assessed by transwell assay in Circ-RAPGEF5 knockdown or overexpression cells. All data are presented as the means ± SD of three independent experiments. *p < 0.05, **p < 0.01, ***p < 0.001, ****p < 0.0001. Figure S3. A Differential expression of SAE1 ICC tumor tissues and adjacent normal tissues in TCGA data. B Kaplan-Meier survival curves of external sequencing data from Dong et al. C qRT-PCR analysis of the relative expression levels of SAE1 in xenografts tissue of groups treated with Sh-Circ-RAPGEF5 and Sh-NC. D Representative IHC images for SAE1 of Sh-NC and Sh-Circ-RAPGEF5 virus treated patient-derived tumor xenograft. E qRT-PCR verified the enrichment efficiency of the Circ-RAPGEF5-biotin probe. F-G qRT-PCR detecting relative SUMO expression in RBE and CCLP1 cells [file 13046_2023_2813_MOESM1_ESM.zip › ADDITIONAL FILE 1/Figure S7.pdf]

A

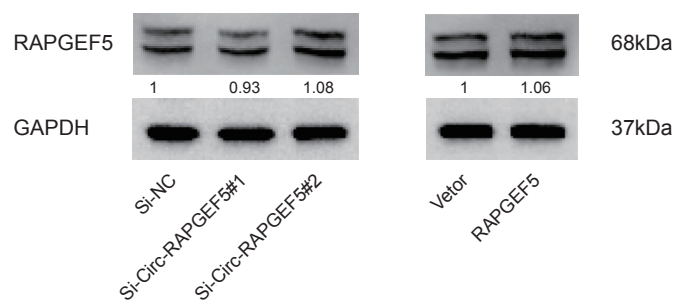

B

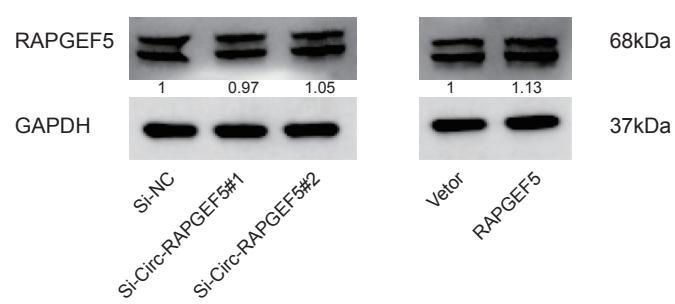

Supplement: Supplementary file 1 — Additional file 1: Table S1. Univariate and multivariate COX regression analysis of the 91 ICC patients. Table S2. Primers used in this study. Table S3. Antibodies and reagents used in this study. Table S4. FISH probes used in this study. Table S5. Biotinylated probes used in this study. Figure S1. The expression levels of Circ-RAPGEF5, SAE1 and miR-3185 in indicated cells. A-B qRT-PCR and western blot analysis detected the expression level of Circ-RAPGEF5 and liner RAPGEF5 in RBE cells after treatment with Si-Circ-RAPGEF5 or Si-NC. C-F The transfected efficiency of Si-Circ-RAPGEF5 and Circ-RAPGEF5 overexpression plasmid in RBE and CCLP1 cells. G-J The transfected efficiency of Si-SAE1 and SAE1 overexpression plasmid in RBE and CCLP1 cells. K-L the overexpression efficiency of miR-3184 mimic in RBE and CCLP1cells. M-N qRT-PCR analysis detected the Circ-RAPGEF5 expression of stably transfected Sh-Circ-RAPGEF5 and Circ-RAPGEF5 RBE cells. *p < 0.05, **p < 0.01, ***p < 0.001, ****p < 0.0001. Figure S2. Circ-RAPGEF5 inhibits apoptosis and promotes migration in ICC cells. A-B Cell apoptosis analysis detected by flow cytometry in Circ-RAPGEF5 knockdown or overexpression cells. C-D The migration ability was assessed by transwell assay in Circ-RAPGEF5 knockdown or overexpression cells. All data are presented as the means ± SD of three independent experiments. *p < 0.05, **p < 0.01, ***p < 0.001, ****p < 0.0001. Figure S3. A Differential expression of SAE1 ICC tumor tissues and adjacent normal tissues in TCGA data. B Kaplan-Meier survival curves of external sequencing data from Dong et al. C qRT-PCR analysis of the relative expression levels of SAE1 in xenografts tissue of groups treated with Sh-Circ-RAPGEF5 and Sh-NC. D Representative IHC images for SAE1 of Sh-NC and Sh-Circ-RAPGEF5 virus treated patient-derived tumor xenograft. E qRT-PCR verified the enrichment efficiency of the Circ-RAPGEF5-biotin probe. F-G qRT-PCR detecting relative SUMO expression in RBE and CCLP1 cells [file 13046_2023_2813_MOESM1_ESM.zip › ADDITIONAL FILE 1/Figure S8.pdf]
